# Mechanistic insights into the ameliorative effects of hypoxia-induced myocardial injury by *Corydalis yanhusuo* total alkaloids: based on network pharmacology and experiment verification

**DOI:** 10.3389/fphar.2023.1275558

**Published:** 2024-01-11

**Authors:** Jiaying Qi, Haoying Li, Yakun Yang, Xiaoqi Sun, Jianxin Wang, Xue Han, Xi Chu, Zhenqing Sun, Li Chu

**Affiliations:** ^1^ School of Pharmacy, Hebei University of Chinese Medicine, Shijiazhuang, Hebei, China; ^2^ The Fourth Hospital of Hebei Medical University, Shijiazhuang, Hebei, China; ^3^ Qingdao Hiser Hospital Affiliated of Qingdao University (Qingdao Traditional Chinese Medicine Hospital), Qingdao, Shandong, China

**Keywords:** *Corydalis yanhusuo* total alkaloids, myocardial ischemia, network pharmacology, apoptosis, oxidative stress, L-type Ca^2+^ currents

## Abstract

**Introduction:** *Corydalis yanhusuo* total alkaloids (CYTA) are the primary active ingredients in *yanhusuo*, known for their analgesic and cardioprotective effects. However, the mechanisms underlying the treatment of Myocardial ischemia (MI) with CYTA have not been reported. The purpose of this study was to explore the protective effect of CYTA on MI and its related mechanisms.

**Methods:** A network pharmacology was employed to shed light on the targets and mechanisms of CYTA’s action on MI. The protective effect of CYTA against hypoxia damage was evaluated in H9c2 cells. Furthermore, the effects of CYTA on L-type Ca^2+^ current (I_Ca-L_), contractile force, and Ca^2+^ transient in cardiomyocytes isolated from rats were investigated using the patch clamp technique and IonOptix system. The network pharmacology revealed that CYTA could regulate oxidative stress, apoptosis, and calcium signaling. Cellular experiments demonstrated that CYTA decreased levels of CK, LDH, and MDA, as well as ROS production and Ca^2+^ concentration. Additionally, CYTA improved apoptosis and increased the activities of SOD, CAT, and GSH-Px, along with the levels of ATP and Ca^2+^-ATPase content and mitochondrial membrane potential. Moreover, CYTA inhibited I_Ca-L_, cell contraction, and Ca^2+^ transient in cardiomyocytes.

**Results:** These findings suggest that CYTA has a protective effect on MI by inhibiting oxidative stress, mitochondrial damage, apoptosis and Ca^2+^ overload.

**Discussion:** The results prove that CYTA might be a potential natural compound in the field of MI treatment, and also provide a new scientific basis for the its utilization.

## 1 Introduction

Ischemic heart disease (IHD) is a serious cardiovascular disease that significantly threatens human health (Liu et al., 2021). In China, the incidence of IHD has been increasing yearly due to changes in lifestyle, dietary habits, an aging population, and other factors. It has now become the second leading cause of death in our population (Wong et al., 2013; Li and Zhang, 2022). Consequently, research on the prevention and treatment of IHD has garnered considerable attention. Currently, various clinical treatment methods are available for IHD, with drug treatment occupying an irreplaceable position. Western medicine has demonstrated remarkable efficacy in treating IHD and is widely used in clinical practice. However, some patients face limitations in its use due to contraindications, adverse drug reactions, and potential toxicity and side effects associated with long-term or combined medication. Therefore, the quest for safe and effective proprietary Chinese medicine for preventing and treating IHD has emerged as a crucial area of medical research (Yang et al., 2019).

The heart, the organ with the highest oxygen utilization and myocardial function demands in the body, is particularly susceptible to myocardial ischemia (MI). During MI, there is limited blood flow supply and an imbalance in oxygen delivery, resulting in severe myocardial hypoxia (Levraut et al., 2003). As such, MI is closely associated with hypoxia (Jin et al., 2023). The primary mechanisms underlying MI-induced damage to cell membranes and mitochondria involve the elevation of reactive oxygen species (ROS) due to hypoxia, intracellular Ca^2+^ overload, and impairment of mitochondrial energy synthesis. Mitochondrial damage serves as both a victim of hypoxia and an initiator of subsequent damage (Chang et al., 2023). The existence of Ca^2+^ is crucial, which is mainly reflected in not only maintaining the normal function of cells, but also participating in cell signal transduction, protein expression and degradation, myocardial contraction, relaxation and other processes (Xue et al., 2021). MI leads to increased permeability of myocardial cell membranes to ions, triggering an influx of Ca^2+^ and causing intracellular Ca^2+^ overload. Consequently, this inhibits mitochondrial respiratory function and induces lysosomal damage and hydrolase release, thereby exacerbating the cellular injury. During MI, mitochondrial oxidative phosphorylation is hindered, impairing ATP synthesis. In severe cases, mitochondrial swelling, crag disintegration, membrane fragmentation, and matrix spillover occur, resulting in irreversible damage (Nunnari and Suomalainen, 2012; Zorov et al., 2014). In addition, Ca^2+^ overload can also increase cell contractility, which in turn induces cardiac hypertrophy and apoptosis (Xue et al., 2021). Therefore, exploring the specific pathogenesis of MI will help to find a more appropriate strategy.

*Yanhusuo* is derived from the dried tubers of the Papaveraceae plant *Corydalis yanhusuo* and is recognized as a blood activator and pain reliever in traditional Chinese medicine. Its primary active components are alkaloids (Ling et al., 2006; Tian et al., 2020). *Yanhusuo* exhibits analgesic and sedative effects and significant therapeutic effects on coronary heart disease, angina pectoris, arrhythmia, and premature beats (Bi et al., 2021). Importantly, it has low toxic side effects and is suitable for long-term use (Tian et al., 2020). *Yanhusuo* extracts have been shown to contain various alkaloids that inhibit apoptosis, thus improving cardiac function (Ling et al., 2006). Among these alkaloids, tetrahydropalmatine (THP) is a notable active compound found in the total alkaloids of Corydalis yanhusuo. In a myocardial infarction model, THP has demonstrated antioxidant and anti-apoptotic properties, offering myocardial protection (Li and Zhang, 2022). So far, the specific mechanism by which CYTA acts on MI is not fully understood. Therefore, in order to find safer and more effective natural drugs to treat MI, it is essential to elucidate the molecular mechanism of action of CYTA.

The H9c2 cells were isolated from rat ventricular tissue, and despite their inability to contract, they still exhibit the functional characteristics of rat cardiomyocytes, rendering them ideal tools for *in vitro* simulation of cardiovascular disease models (Fang et al., 2018; Vanacore et al., 2018). Meanwhile, CoCl_2_, a widely employed hypoxia inducer, has been extensively utilized for *in vitro* simulation of hypoxia/ischemia injury (Pecoraro et al., 2018; Han et al., 2022). Referred to the aforementioned theories, we hypothesized that CYTA might have a protective effect against MI. Therefore, in order to investigate the protective mechanism of CYTA against MI, a comprehensive network pharmacology approach was first performed to analyze the key proteins, pathways and mechanisms involved in the treatment of MI by CYTA. To experimentally validate these findings, H9c2 cells were employed as the experimental model, specifically utilizing the CoCl_2_-induced hypoxia model. Furthermore, the effects of CYTA on L-type Ca^2+^ current (I_Ca-L_), cell contractility, and Ca^2+^ transients were further investigated using acute isolated rat ventricular myocytes. By delving into the underlying mechanisms, this study aims to offer a more thorough understanding of how CYTA treats MI.

## 2 Materials and methods

### 2.1 Network pharmacology analysis

The alkaloid components of *Yanhusuo* were obtained from The Traditional Chinese Medicine Systems Pharmacology Database (TCMSP, https://old.tcmsp-e.com/index.php), along with literature databases. The collection of targets has been completed. The TCMSP database was searched with “*yanhusuo*” as the keyword, and all the alkaloid components were recorded in the results by selecting the classification of “ingredients.” Subsequently, the detailed information of each alkaloid was checked, its related targets were recorded, and the duplicate values were removed, and the results were used as CYTA-related targets. The gene names corresponding to all target proteins were standardized using the UniProtKB database (https://www.uniprot.org/). The GeneCards database (https://www.genecards.org/) was searched for relevant targets with the keyword “myocardial ischemia,” and all the target results were exported as MI-related genes.

The targets of CYTA and MI were inputted into the online Venn analysis tool (https://bioinfogp.cnb.csic.es/tools/venny/) to identify overlapping targets to be used as anti-MI targets for CYTA. The collected target information was then imported into the STRING database (https://string-db.org/), with the attribute set as *Homo sapiens* and the threshold set to 0.4, resulting in the retrieval of a protein interaction network (PPI) in TSV format. Subsequently, the files were imported into Cytoscape 3.6.1 to construct the PPI network diagram. The Network Analyzer tool within the software was utilized to analyze the topological parameters of the network interaction graph, such as degree and betweenness centrality (BC), which indicate the degree of correlation between nodes in the network graph. By applying parameter values, key nodes in the network graph were identified, leading to the investigation of CYTA’s mechanism on MI. Finally, the intersecting target genes underwent protein molecular function (MF), cell population (CC), biological process (BP), and KEGG analysis, which were performed using Metascape (https://metascape.org/gp/index.html). Throughout the analysis, a confidence level of *p* < 0.01 was employed.

### 2.2 Drugs and reagents

CYTA (purity ≥90%), purchased from Chengdu Dexter Biotechnology Co., LTD., was synthesized and dissolved in dimethyl sulfoxide (DMSO) as 100 mM stock solutions. It was diluted to a specific concentration when used. Cell Counting Kit-8 (CCK-8) was provided by Beijing Zhuo Man Biotechnology Co. in Beijing, China. Type II collagenase was obtained from Worthington Biochemical in Lakewood, United States. Isoprenaline (ISO) and verapamil (VER) were supplied by Hefeng Pharmaceutical Co. in Shanghai, China. Unlabeled reagents of analytical purity were obtained from Sigma Chemical Co. in Missouri, United States. All experiment kits, including CK (catalog: A0321-1), LDH (catalog: A020-1-2), SOD (catalog: A001-3-2), CAT (catalog: A007-1), MDA (catalog: A020-1-2), GSH-Px (catalog: A005-1-2), ATP (catalog: A095-11), Ca^2+^-ATPase (catalog: A016-1-2), and BCA (catalog: A045-3), were purchased from Jiancheng BioEngineering Institute in Nanjing, China.

### 2.3 Cell culture and treatment

H9c2 cells (Bluefbio, Shanghai, China; catalog: BFN60804388) were cultured in High-sugar Dulbecco’s Modified Eagle Medium (DMEM) (Gibco; Thermo Fisher Scientific, Waltham, United States) supplemented with 10% inactivated fetal bovine serum (FBS) (Gibco; Thermo Fisher Scientific, Waltham, United States) and 100 U/mL penicillin/streptomycin (Leagene Biotechnology; Beijing, China). The incubator maintained a stable temperature (37°C), CO_2_ level (5%), pH (7.2–7.4), and high relative humidity (95%). Cells reached 80% confluency or higher after 2–3 days of growth in culture flasks. After washing with Phosphate-buffered saline (PBS) (Solarbio Life Sciences; Beijing, China), the cells were detached using 0.25 g/L trypsin (Leagene Biotechnology; Beijing, China). Cells with appropriate density were uniformly seeded in 96-well plates, 24-well plates, or 6-well plates for subsequent experiments.

### 2.4 Model establishment and experimental design

H9c2 cells were exposed to various concentrations of CoCl_2_ (200, 400, 600, 800, and 1,000 μM) for 22 h and different concentrations of CYTA (1, 3, 10, 30, 100, and 300 μg/mL) to assess their impact on cell viability and determine the optimal concentrations of CoCl_2_ and CYTA. At the end of the treatment, 10 μL of CCK-8 was added to each well to measure the absorbance value. Based on the aforementioned procedure, the optimal pretreatment concentration and time for CYTA were determined as 10 and 30 μg/mL for 4 h, respectively. The optimal concentration of CoCl_2_ was found to be 800 μM. The experimental groups were categorized as follows: 1) Control (Con); 2) CoCl_2_; 3) CoCl_2_ + L-CYTA; 4) CoCl_2_ + H-CYTA.

### 2.5 Assessment of biochemical analysis

After the treatment, the supernatant was collected to measure the release of CK and LDH, following the instructions provided with the kit. H9c2 cells were scraped and centrifuged to obtain the cell pellet. The pellet was resuspended in an appropriate amount of PBS and sonicated for fragmentation. The protein concentrations of each cell group were determined using the BCA kit and used for subsequent assays of SOD, MDA, CAT, GSH-Px, ATP, and Ca^2+^-ATPase, following the instructions provided with the respective kits.

### 2.6 Assessment of ROS levels

Following the treatment, the medium was aspirated, and H9c2 cells cultured in 24-well plates were washed with PBS. Next, 500 μL of 2,7-dichlorodihydrofluorescein diacetate (DCFH-DA, Cayman Chemical Company, Michigan, United States) was added to each well and incubated for 20 min in a dark environment. Subsequently, the remaining dye was washed off with PBS, and fluorescence images were captured using a fluorescence microscope. The acquired images were analyzed quantitatively using Image-Pro Plus 6.0 software (Media Cybernetics, Inc.).

### 2.7 Assessment of Ca^2+^

Following the treatment of H9c2 cells in 24-well plates, the plates were washed with PBS. Subsequently, 500 μL of 10 μmol/L Fluo-3 AM dye (Life Technologies, Carlsbad, United States) was added and incubated for 20 min in a light-free environment. After washing off the excess dye with PBS, fluorescent images were captured using a fluorescence microscope. The acquired images were subjected to quantitative analysis using Image-Pro Plus 6.0 software.

### 2.8 Assessment of mitochondrial membrane potential

After treating the cells in 24-well plates, 500 μL of Rhodamine 123 (Rh123, Yuanye Biotechnology, Shanghai, China) dye was added to each well. The cells were then incubated at 37°C in the dark for 20 min. Subsequently, the remaining dye in each well was washed off with PBS, and fluorescence images were captured using a fluorescence microscope. The acquired images were subjected to quantitative analysis using Image-Pro Plus 6.0 software.

### 2.9 Assessment of apoptosis

Following the treatment of cells cultured in 24-well plates, 500 μL of Hoechst-33258 staining solution (Beijing Solarbio Science & Technology, Beijing, China; Catalog: IH0060) at a concentration of 10 μg/mL was added to each well. The cells were then incubated in the dark at 37°C for 20 min. Subsequently, the residual dye in the cells was washed off with PBS. The cells were placed under a fluorescence microscope to capture fluorescence images. Finally, the acquired images were analyzed quantitatively using Image-Pro Plus 6.0 software.

### 2.10 Experimental animals

Forty male Sprague-Dawley (SD) rats aged 6–8 weeks and weighing 200 ± 20 g were obtained from the Experimental Animal Centre of Hebei Medical University. The rats were housed in a controlled environment with an ambient temperature of 21°C ± 1°C and a humidity level of 40%–60%. Rats were housed under 12/12 h light/dark cycle and up to five animals per cage. They had free access to food and water and were acclimated for 1 week before the experiments. All procedures followed the ethical guidelines for experimental animals set by the Hebei University of Traditional Chinese Medicine (approval number: 2103062). The rats were randomly divided into 2 groups: CON and ISO. To establish the myocardial infarction (MI) model, each rat received subcutaneous injections of ISO for two consecutive days (85 mg/kg/day) (Hosseini et al., 2022; Wahid et al., 2022). Subsequently, cardiomyocytes were acutely isolated from a single rat at a specific time point. This study was carried out following the recommendations of the Declaration of Helsinki. Animal experiments and methods were performed in accordance with the National Institutes of Health Guidelines for the Care and Use of Laboratory Animals.

### 2.11 Preparation of ventricular cardiomyocytes

The rats were anesthetized by intraperitoneal injection of sodium heparin (500 IU/KG) for 15 min, followed by an intraperitoneal injection of ethyl carbamate (1.0 g/KG). After the anesthesia was completed, the rat’s thorax was opened to separate the aorta. The heart was quickly removed and placed in a Ca^2+^-free Tyrode ice solution, and the cardiac aorta was inserted into the Langendorff device. The Ca^2+^-free Tyrode solution was injected into the heart for 5 min in a thermostatic device at 37°C. The prepared enzyme solution was then perfused recurrently for 15–20 min to digest the myocardium, followed by rinsing with Ca^2+^-free Tyrode solution to terminate digestion. Subsequently, the heart was placed in the Kreb’s buffer, and the ventricular muscle was quickly torn to yield a final KB suspension of individual ventricular myocytes. This suspension was left to stand for 1 h at 4°C before the remaining procedures were conducted. The optimal time for the cells to be available is 8 h. All solutions used were continuously oxygenated at a perfusion rate of 4 mL/min to ensure a 100% oxygen content. The configuration details of the solutions used are presented in Table 1.

**TABLE 1:** Method of preparation of the solution.

Solution components (mM)	Enzyme solution	Tyrode’s solution	External solution	Internal solution	Kreb’s buffer
Hepes free acid (HEPES)	10	10	10	10	10
Glucose	10	10	10		10
CaCl_2_	0.03	1.8	1.8		
MgCl_2_	1	1	2		
Taurine	4.4	10			20
NaCl	135	135			
KCl	5.4	5.4			
NaH_2_PO_4_	0.33	0.33			
Bovine serum albumin	0.5^a^				
Collagenase type II	0.6^a^				
Tetraethylammonium chloride (TEA-Cl)			140	20	
CsCl				120	
Mg-ATP				5	
EGTA				10	1
MgSO_4_					3
KCl					40
KH_2_PO_4_					25
KOH					80
Glutamic acid					50

Using NaOH, to adjust the pH of the enzyme solution and normal Tyrode’s solution to 7.4. In contrast to normal Tyrode’s solution, Ca^2+^-free Tyrode’s solution contains no CaCl_2_ and taurine. Using CsOH, and KOH, the pH of the inteikrnal and external solutions and Kreb’s buffer solutions were adjusted to 7.3 and 7.2, respectively.

^a^ Unit in mg/mL.

### 2.12 Electrophysiological recording

A suspension of ventricular myocytes was transferred to the cell recording chamber of the membrane clamp. Once the cells became stable and attached to the bottom of the recording chamber, an external solution of cells was added to suppress any interference from other currents. Cells with intact and clean edges, free from surface particles, clear striations, and at rest, were selected for the experiment. The whole-cell membrane clamp recording method was employed to hold the cells in place. After zeroing the internal and external potential difference, a borosilicate glass electrode (filled with internal solution, resistance 3–5 MΩ) was applied to the cell membrane and then sealed. The recorded streams with a 2 kHz filter were amplified using an Axopatch 200B amplifier (Axon Instruments, Union City, CA, United States) and analyzed using pCLAMP 10.2 software (protocol: pulse waveform of 10 mV, 1 ms; frequency duration of 10 Hz).

### 2.13 Contractility and Ca^2+^ transient measurement

To assess cardiomyocyte contractility, the cell suspension was gently introduced into the cell bath of the microscope. While the cells settled at the bottom of the bath, a calcium-free solution was perfused through the cells at a rate of 1 mL/min. Subsequently, the cardiomyocytes were stimulated to contract regularly at a frequency of 0.5 Hz (duration 2 ms) using a cell stimulator (MyoPacer, IonOptix), and the cell contractions were continuously recorded without any treatment using the IonOptix Myocardial Assay System (IonOptix, Milton, MA, United States). Following the recordings, the cells were returned to regular solution conditions.

Cardiomyocyte Ca^2+^ transients were recorded by observing changes in the fluorescent indicator. The cardiomyocytes were incubated with the fluorescent Ca^2+^ indicator, fluo2-AM (2 mM), for a duration of 10 min. The cell suspensions were then gently added to the bath, and cell excitation was induced by applying electric field stimulation through a sheet platinum electrode at a frequency of 0.5 Hz and a duration of 2 ms. The light from a 75 W UV xenon lamp was modulated by the electric field stimulation through a 340 or 380 nm filter to reach the ventricular myocytes, causing them to emit 510 nm light. The resulting fluorescence signal was examined using the IonOptix system.

### 2.14 Data analysis and statistics

The resulting experimental data were subjected to a one-way analysis of variance (ANOVA) followed by Tukey’s *post hoc* test using Origin 9.1 software. The results were expressed as mean ± standard error (SEM). A *p*-value of less than 0.05 was considered statistically significant.

## 3 Results

### 3.1 Network pharmacology analysis of potential targets and mechanisms of CYTA against MI

**FIGURE 1:**
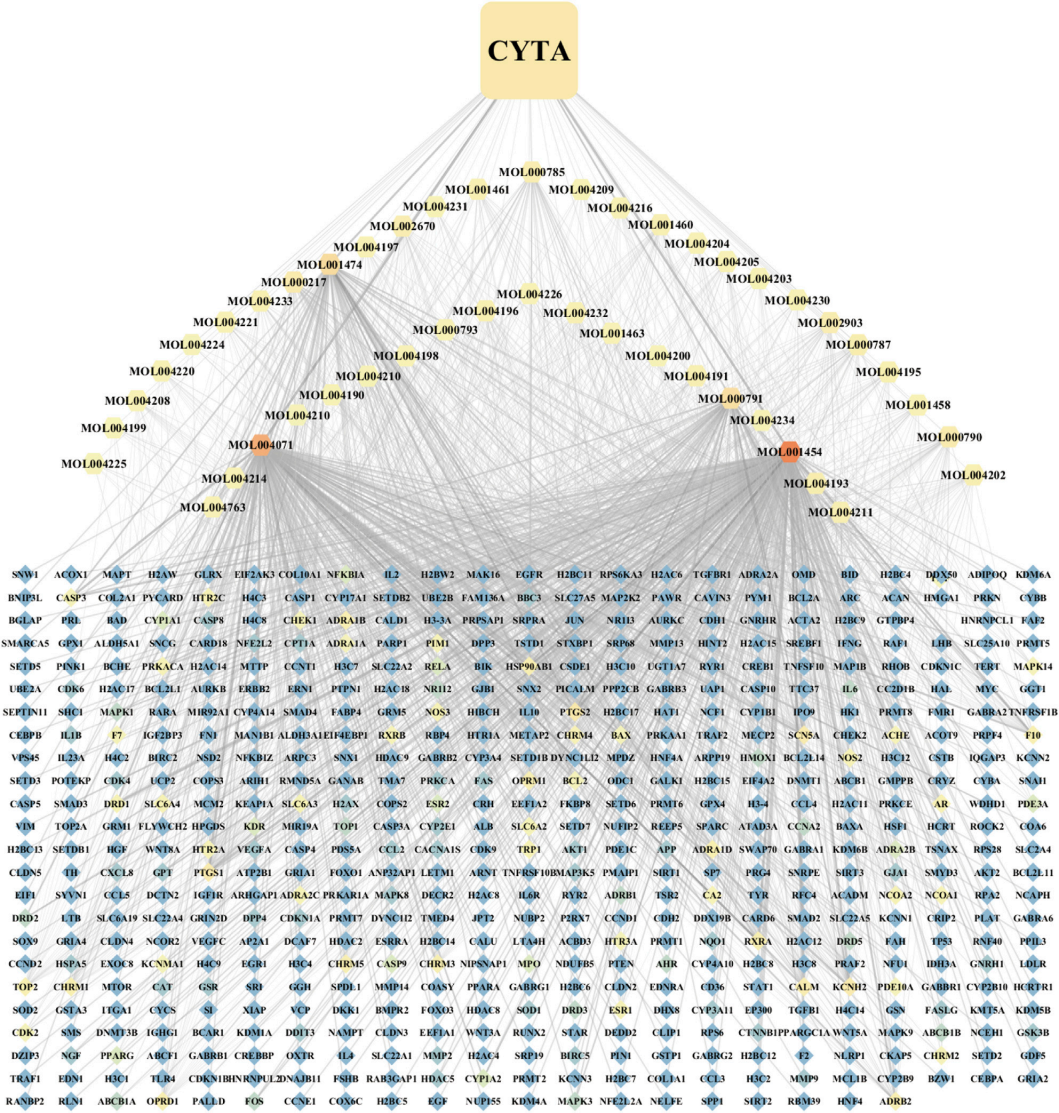
Component-target network diagram of CYTA.

**FIGURE 2:**
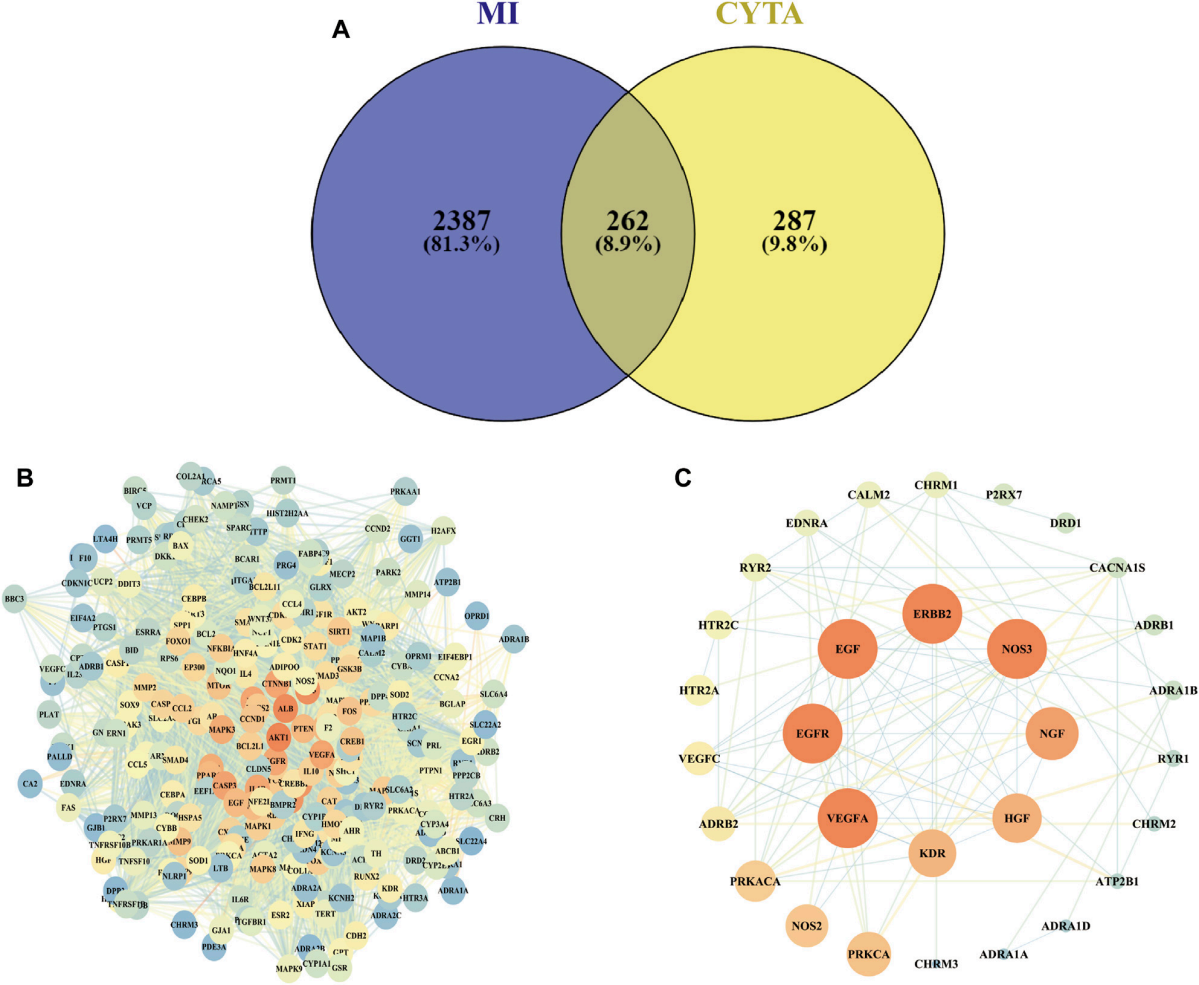
Target screening for CYTA against MI **(A)** The number of intersections between CYTA and MI targets. **(B)** PPI network diagram based on CYTA and MI intersection targets. **(C)** The targets are related to the calcium signaling pathway.

A comprehensive search was conducted on a total of 46 CYTA components using the TCMSP database and relevant literature (Table 2). Figure 1 illustrates the 549 targets associated with each component of CYTA retrieved from the TCMSP database. The MI-related genes were identified from the GeneCards database. Duplicate targets were removed, resulting in a total of 2649 MI-related targets. The Venn diagram (Figure 2A) demonstrates the intersection of potential targets between CYTA and MI, revealing 262 identical genes. To further examine the relationship among these 262 genes, the PPI network (Figure 2B) was generated using Cytoscape 3.6.1. The network represents proteins as nodes and their relationships as connecting lines, where nodes and connectors withhigh degree and BC values tend to be brighter in color and *vice versa*.

**TABLE 2:** OB, DL values and molecule IDs for the 46 active ingredients of CYTA.

Mol ID	Molecule name	OB(%)	DL
MOL004071	Hyndarin	73.94	0.64
MOL004195	CORYDALINE	65.84	0.68
MOL000785	palmatine	64.6	0.65
MOL000787	Fumarine	59.26	0.83
MOL002903	(R)-Canadine	55.37	0.77
MOL004230	stylopine	48.25	0.85
MOL004203	Dehydrocorybulbine	46.97	0.63
MOL004205	Dehydrocorydalmine	43.9	0.59
MOL004204	dehydrocorydaline	41.98	0.68
MOL004216	13-methylpalmatrubine	40.97	0.63
MOL001454	berberine	36.86	0.78
MOL004209	13-methyldehydrocorydalmine	35.94	0.63
MOL000790	Isocorypalmine	35.77	0.59
MOL001458	coptisine	30.67	0.86
MOL004211	Glauvent	29.03	0.61
MOL004193	Clarkeanidine	86.65	0.54
MOL001460	Cryptopin	78.74	0.72
MOL004234	2,3,9,10-tetramethoxy-13-methyl-5,6-dihydroisoquinolino [2,1-b] isoquinolin-8-one	76.77	0.73
MOL000791	bicuculline	69.67	0.88
MOL004191	Capaurine	62.91	0.69
MOL004200	methyl-[2-(3,4,6,7-tetramethoxy-1-phenanthryl)ethyl]amine	61.15	0.44
MOL001463	Dihydrosanguinarine	59.31	0.86
MOL004232	tetrahydroprotopapaverine	57.28	0.33
MOL004226	Pseudoprotopine	53.75	0.83
MOL004196	Corydalmine	52.5	0.59
MOL000793	Bulbocapnine	47.54	0.69
MOL004198	Corynoline	46.06	0.85
MOL004210	(1S,8′R)-6,7-dimethoxy-2-methylspiro [3,4-dihydroisoquinoline-1,7′-6,8-dihydrocyclopenta[g][1,3]benzodioxole]-8′-ol	43.95	0.72
MOL004220	N-methyllaurotetanine	41.62	0.56
MOL004214	isocorybulbine	40.18	0.66
MOL004763	Izoteolin	39.53	0.51
MOL004202	dehydrocavidine	38.99	0.81
MOL004208	demethylcorydalmatine	38.99	0.54
MOL004225	pseudocoptisine	38.97	0.86
MOL004199	Corynoloxine	38.12	0.6
MOL001474	sanguinarine	37.81	0.86
MOL004197	Corydine	37.16	0.55
MOL002670	Cavidine	35.64	0.81
MOL004231	Tetrahydrocorysamine	34.17	0.86
MOL001461	Dihydrochelerythrine	32.73	0.81
MOL000217	(S)-Scoulerine	32.28	0.54
MOL004233	(+)-Thaliporphine	31.87	0.56
MOL004221	norglaucing	30.35	0.56
MOL004224	pontevedrine	30.28	0.71
MOL004290	(−)-alpha-N-methylcanadine	45.06	0.8
MOL004228	saulatine	42.74	0.79

**FIGURE 3:**
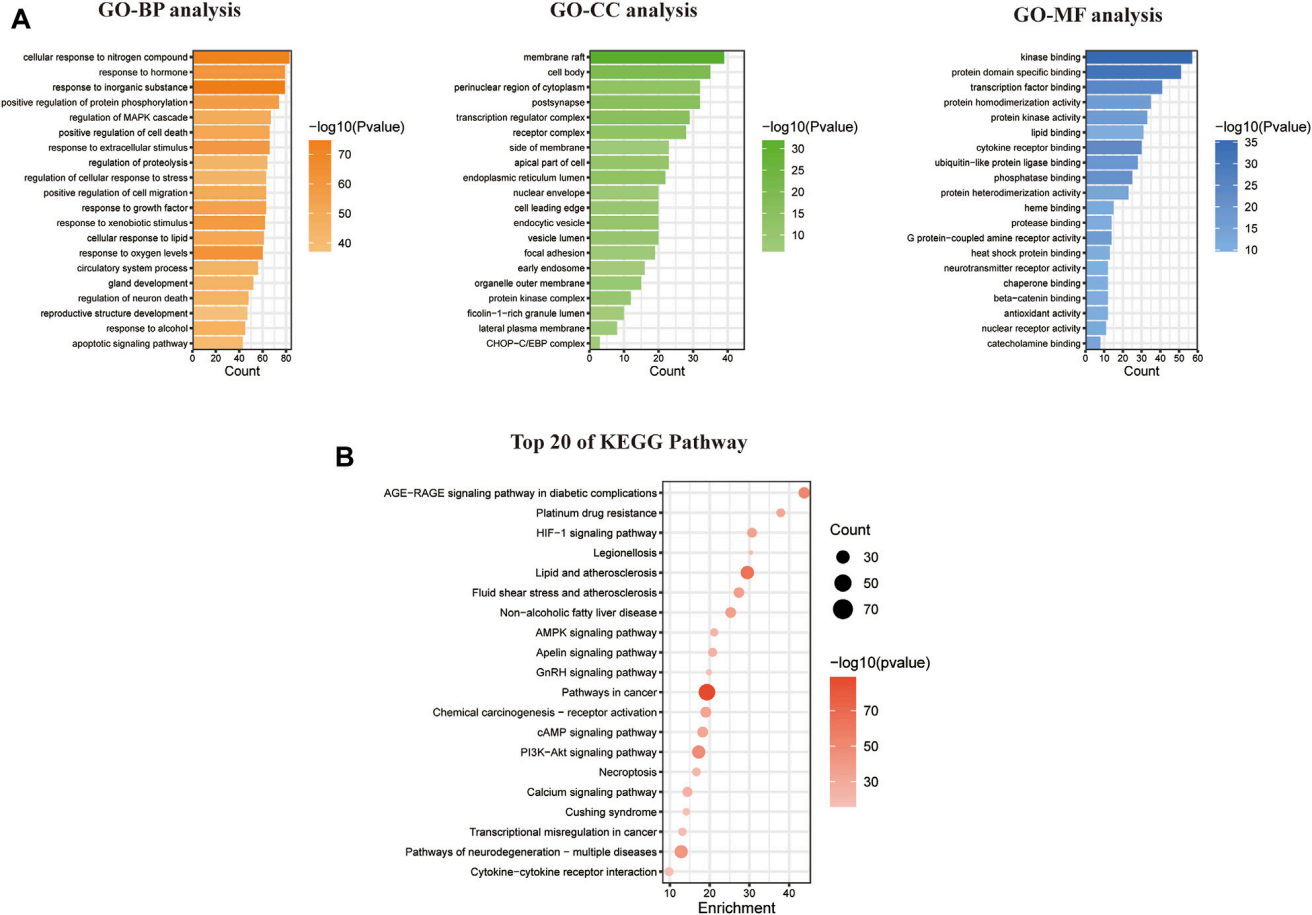
Enrichment analysis of GO and KEGG pathways based on CYTA and MI intersection targets. **(A)** GO analysis graphs of potential targets. **(B)** Enrichment analysis of the KEGG pathway for potential targets.

The GO enrichment analysis yielded 185 BP items, 130 CC items, and 108 MF items. The BP analysis highlighted topics such as positive regulation of cell death, response to oxygen levels, regulation of neuron death, and apoptotic signaling pathways. Regarding CC, the targets were primarily associated with the cell membrane, nucleus, mitochondria, and endoplasmic reticulum. The MF analysis revealed that the intersecting targets were linked to kinase binding, protein domain-specific binding, and transcription factor binding, as depicted in Figure 3A.

In relation to the KEGG enrichment analysis of CYTA for MI, it identified 174 pathways. Further analysis of these pathways showed a strong association with apoptosis and the calcium signaling pathway. The top 20 results, based on the *p*-value, were selected and presented in a bubble diagram (Figure 3B). The results indicate the involvement of apoptosis, mitochondrial energy metabolism, and oxidative stress in the preventive effects of CYTA against MI. Additionally, based on the KEGG enrichment analysis, numerous targets were found to be closely associated with the calcium signaling pathway. Therefore, we extracted targets associated with the calcium signaling pathway (Figure 2C), where nodes and connecting lines with high degree values and BC tended to be larger and brighter in color, and *vice versa*.

### 3.2 Effects of different concentrations of CYTA in H9c2 cells viability

**FIGURE 4:**
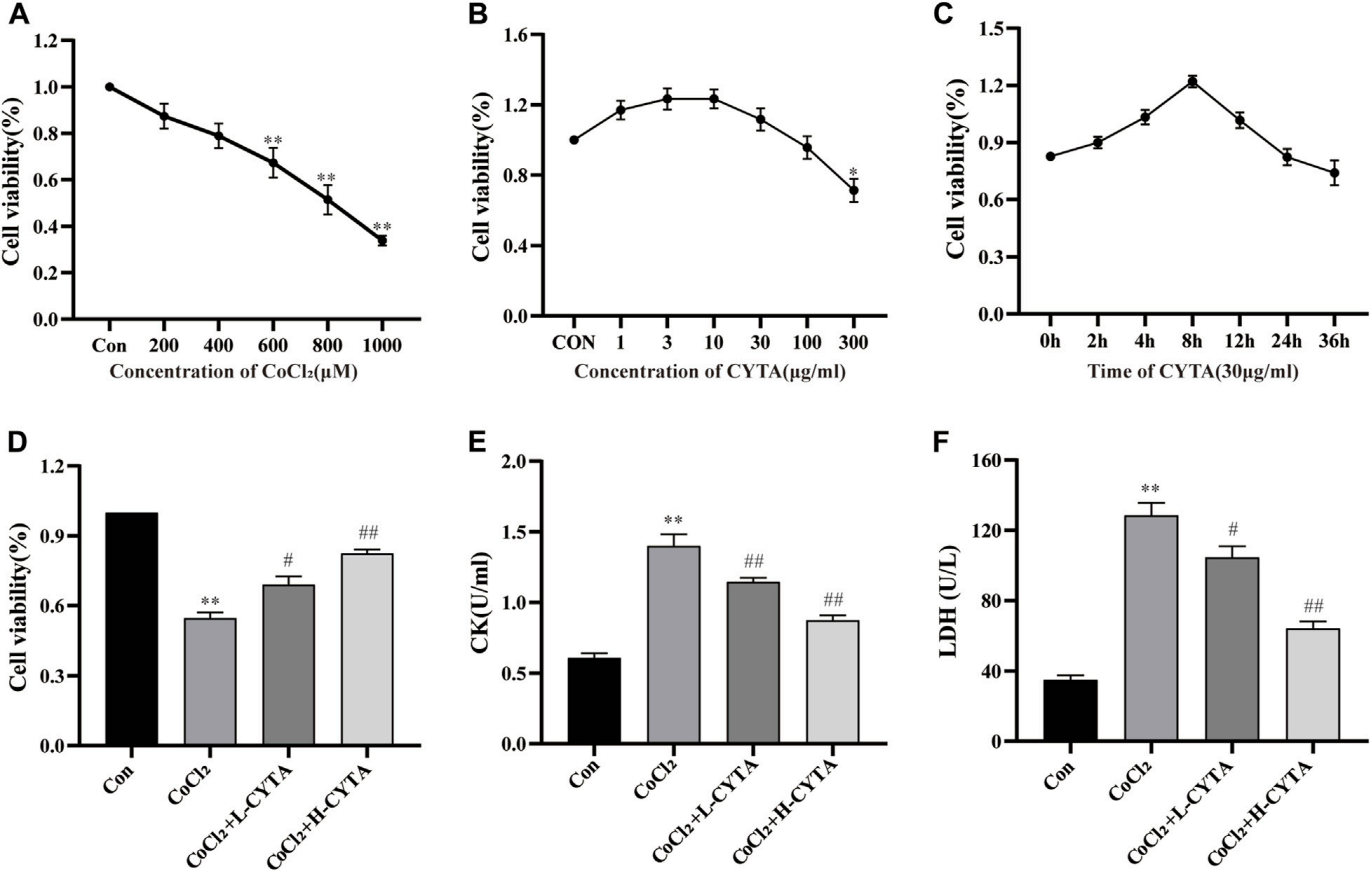
Determination of CoCl_2_ and CYTA concentrations and the protective effects of CYTA on cardiac enzymes in hypoxic H9c2 cells. **(A)** H9c2 cells were incubated with CoCl_2_ (200–1,000 μM) alone for 22 h **(B)** H9c2 cells were incubated with CYTA (1–300 μg/mL) alone for 24 h **(C)** Cells were incubated with CYTA (30 μg/mL) alone for 0–36 h **(D)** The protective effect of CYTA on H9C2 cells damaged by CoCl_2_, CYTA was selected at 10 and 30 μg/mL for 4 h, CoCl_2_ was selected at 800 μM. **(E,F)** The effects of CYTA treatment on LDH and CK levels in CoCl_2_-induced H9c2 cell damage. Mean ± SEM, *n* = 3. *p < 0.05, **p < 0.01 versus CON group and #*p* < 0.05, ##*p* < 0.01 versus CoCl_2_ group. Note: L-CYTA: 10 μg/mL and H-CYTA: 30 μg/mL.

According to Figure 4B, concentrations of 1–100 μg/mL of CYTA did not exhibit significant inhibition of normal H9c2 cell proliferation (*p* > 0.05). However, at a 300 μg/mL concentration, an inhibition of normal H9c2 cell proliferation was observed (*p* < 0.01). Furthermore, at a concentration of 30 μg/mL, no inhibition of H9c2 cells was observed from 0 to 36 h (*p* > 0.05, Figure 4C). Based on these findings, dosing concentrations of 10 μg/mL and 30 μg/mL were selected.

### 3.3 Effect of CYTA in hypoxic H9c2 cells viability

The concentration of CoCl_2_ that induced hypoxia was determined to be 800 μmol/L, as depicted in Figure 4A. Figure 4D shows that the survival of H9c2 cells was significantly reduced in the CoCl_2_ group compared to the CON group (*p* < 0.01). However, pretreatment with either 10 or 30 μg/mL CYTA demonstrated a mitigating effect on hypoxic injury (*p* < 0.05 or 0.01).

### 3.4 Effects of CYTA on CK and LDH levels in hypoxic H9c2 cells

Figures 4E, F demonstrate that the release of CK and LDH into the extracellular medium was significantly elevated in the CoCl_2_ group compared to the CON group (*p* < 0.01). In contrast, CYTA exhibited the ability to significantly decrease the levels of CK and LDH in the supernatant of hypoxic H9c2 cells (*p* < 0.05 or 0.01), indicating a mitigating effect of CYTA on cell injury.

### 3.5 Effects of CYTA on ROS levels in hypoxic H9c2 cells

**FIGURE 5:**
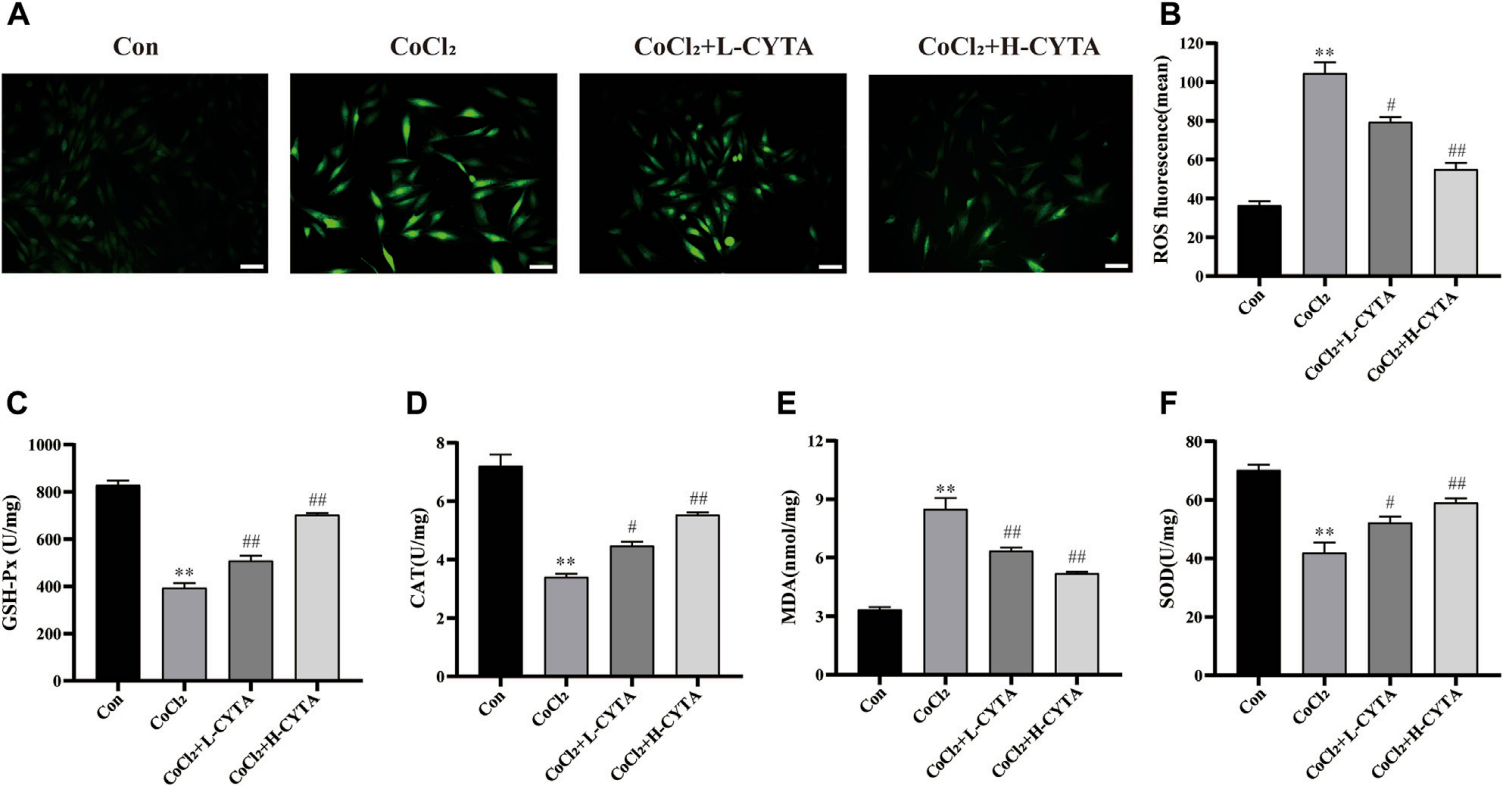
Effects of CYTA on intracellular ROS levels and oxidative stress indicators in hypoxic H9c2 cells. **(A)** Intracellular ROS levels observed under fluorescence microscopy (scale bar: 100 μm; magnification: ×200). Intense green fluorescence was displayed when intracellular ROS levels increased. **(B)** Results of the quantitative analysis of the mean fluorescence intensity of ROS. **(C–F)** Effect of CYTA on intracellular GSH-Px, CAT, MDA and SOD levels. Mean ± SEM, *n* = 3 or 6. *p < 0.05, **p < 0.01 versus CON group and ^#^ *p* < 0.05, ^##^ *p* < 0.01 versus CoCl_2_ group. Note: L-CYTA: 10 μg/mL and H-CYTA: 30 μg/mL.

The results obtained from the measurement of ROS can be interpreted such that a higher average fluorescence intensity value corresponds to increased ROS production in the cells. Compared to the normal group, the fluorescence intensity of ROS in H9c2 cells was significantly elevated in the CoCl_2_ group (*p* < 0.01). However, in both the high and low-dose groups of CYTA, the fluorescence intensity of ROS in H9c2 cells was significantly reduced compared to the CoCl_2_ group (*p* < 0.05 or 0.01). The results are depicted in Figures 5A, B.

### 3.6 Effects of CYTA on oxidative stress homeostasis in hypoxic H9c2 cells

As depicted in Figures 5C–F, the activities of SOD, GSH-Px, and CAT were decreased, while the activity of MDA was increased in the CoCl_2_ group compared to the CON group (*p* < 0.01). Conversely, in the CYTA group, the activities of SOD, GSH-Px, and CAT were increased, and the level of MDA was decreased in the supernatant compared to the CoCl_2_ group (*p* < 0.05 or 0.01).

### 3.7 Effects of CYTA on mitochondrial membrane potential in hypoxic H9c2 cells

**FIGURE 6:**
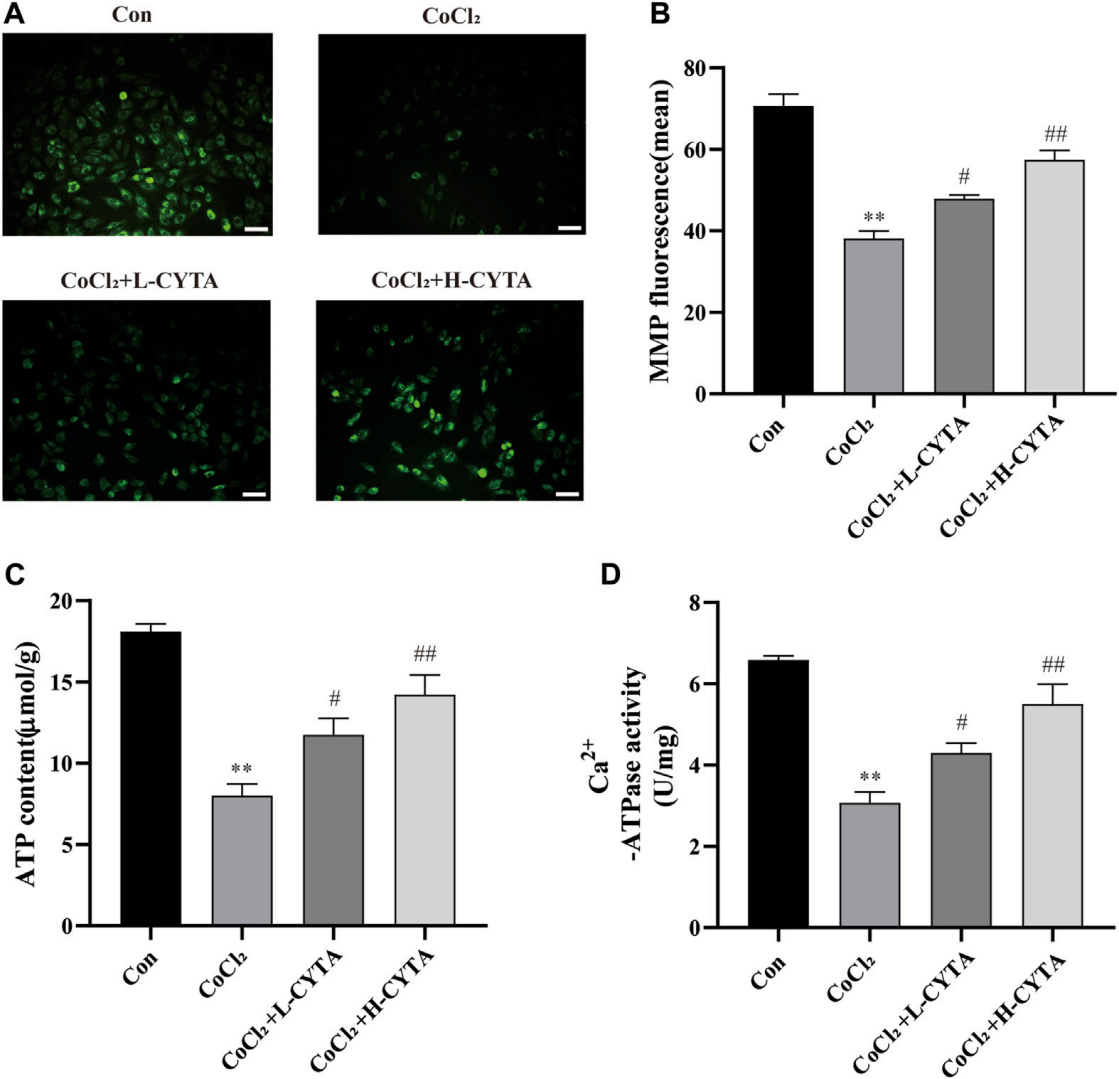
Effects of CYTA on MMP in hypoxic H9c2 cells. **(A)** Changes in the MMP of cells observed under fluorescence microscopy (scale bar: 100 μm; magnification: ×200). When the mitochondrial membrane potential was lost, the yellow-green fluorescence intensity within the mitochondria was significantly reduced. **(B)** Results of quantitative analysis of the mean fluorescence intensity of MMP **(C)** ATP content and **(D)** Ca^2+^-ATPase activity were detected. Mean ± SEM, *n* = 6. *p < 0.05, **p < 0.01 versus CON group and ^#^ *p* < 0.05, ^##^ *p* < 0.01 versus CoCl_2_ group. Note: L-CYTA: 10 μg/mL and H-CYTA: 30 μg/mL.

As shown in Figures 6A, B, damage to the mitochondrial membrane resulted in a decrease in membrane potential. Rh123 fluorescence staining allowed the detection of changes in intracellular mitochondrial membrane potential. Compared to the CON group, the mitochondrial membrane potential of H9c2 cells was significantly reduced after hypoxic injury, and the difference was statistically significant (*p* < 0.01). However, after pretreatment with CYTA, the reduction in mitochondrial membrane potential was significantly improved in H9c2 cells compared to the hypoxic injury group (*p* < 0.05 or 0.01).

Both ATP content and Ca^2+^-ATPase activity are direct and indirect indicators of mitochondrial function. Treatment with CoCl_2_ significantly reduced the ATP content and Ca^2+^-ATPase activity in H9c2 cells (*p* < 0.01). However, pretreatment with CYTA resulted in varying degrees of increased intracellular ATP content and Ca^2+^-ATPase activity in H9c2 cells (*p* < 0.05 or 0.01) (Figures 6C, D).

### 3.8 Effects of CYTA on apoptosis in hypoxic H9c2 cells

**FIGURE 7:**
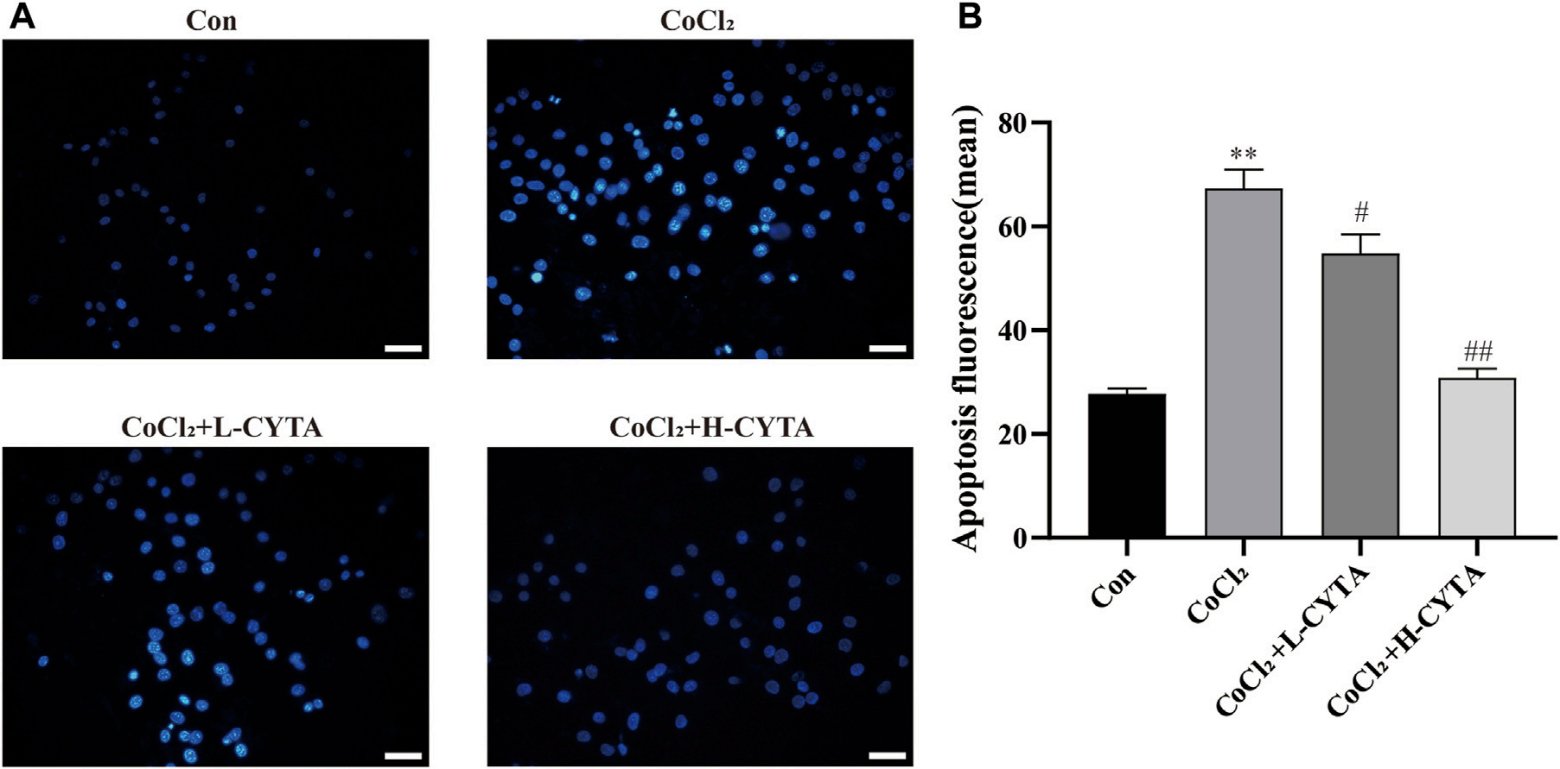
Effects of CYTA on apoptosis in hypoxic H9c2 cells. **(A)** Levels of apoptosis in H9c2 cells observed under fluorescence microscopy (scale bar: 100 μm; magnification: ×200). When cells apoptosis, the nuclei are bright blue fluorescence. **(B)** Results of quantitative analysis on the mean fluorescence intensity of apoptotic cells. Mean ± SEM, *n* = 3. *p < 0.05, **p < 0.01 versus CON group and ^#^ *p* < 0.05, ^##^ *p* < 0.01 versus CoCl_2_ group. Note: L-CYTA: 10 μg/mL and H-CYTA: 30 μg/mL.

Under fluorescence microscopy, the CON group showed no obvious apoptotic cells, with nuclei appearing light blue and having low fluorescence. In the CoCl_2_ group, the nuclei exhibited uneven coloring, and apoptotic cells appeared bright blue with high fluorescence (*p* < 0.01). After CYTA treatment, the number of apoptotic cells gradually decreased (Figure 7A, *p* < 0.05 or 0.01). The apoptotic status of each cell group is depicted in Figure 7B.

### 3.9 Effects of CYTA on Ca^2+^ content in hypoxic H9c2 cells

**FIGURE 8:**
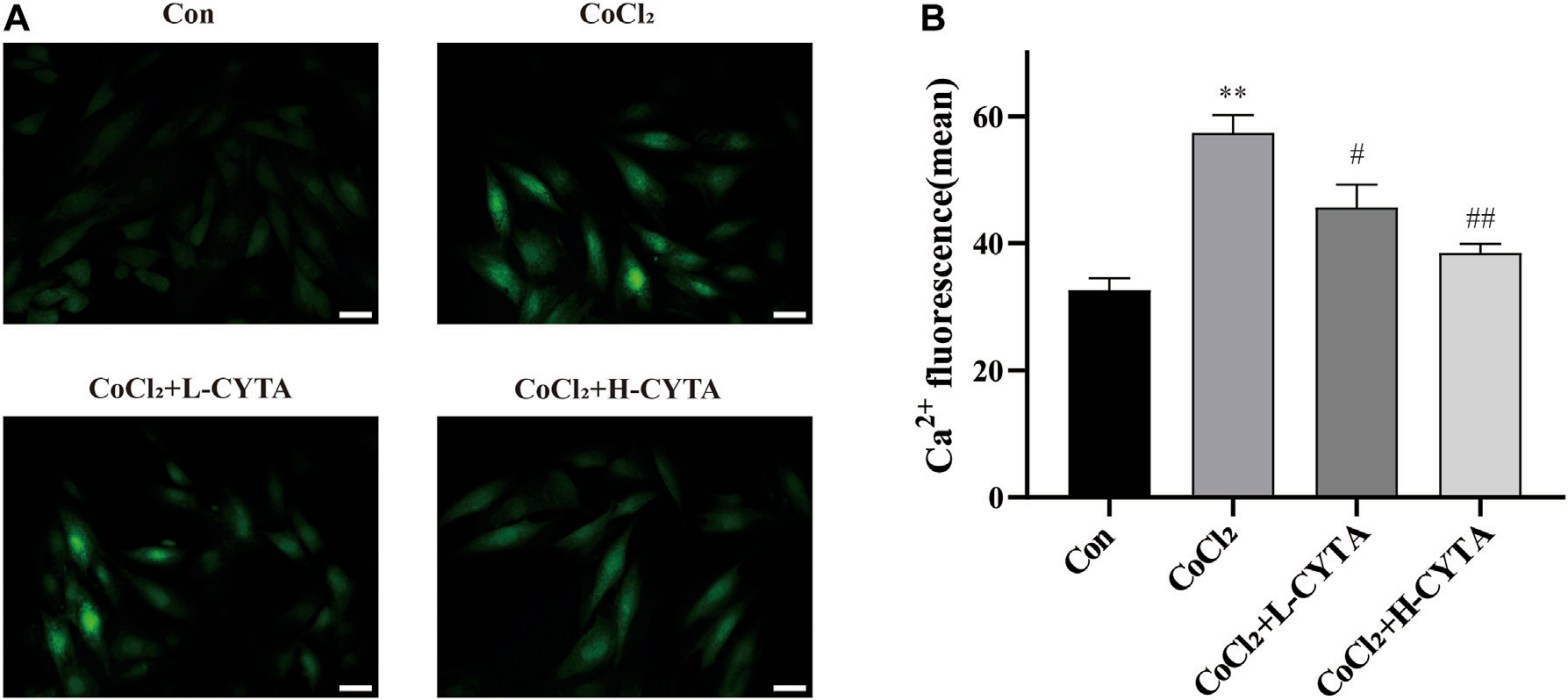
Effects of CYTA on intracellular Ca^2+^ concentration in hypoxic H9c2 cells. **(A)** Changes in intracellular Ca^2+^ concentration observed under fluorescence microscopy (scale bar:100 μm; magnification: ×200). The fluorescence changes became stronger when Fluo-3 combined with Ca^2+^. **(B)** Results of the quantitative analysis on the mean fluorescence intensity of Ca^2+^. Mean ± SEM, *n* = 3. *p < 0.05, **p < 0.01 versus CON group and ^#^ *p* < 0.05, ^##^ *p* < 0.01 versus CoCl_2_ group. Note: L-CYTA: 10 μg/mL and H-CYTA: 30 μg/mL.

The results are depicted in Figure 8. It is evident that CoCl_2_ significantly increased the cytoplasmic Ca^2+^ content in H9c2 cells (*p* < 0.01), while pretreatment with CYTA significantly attenuated the increase in Ca^2+^ content (*p* < 0.01 or 0.05). These findings suggest that CYTA can regulate and maintain normal Ca^2+^ content in the cytoplasm of cardiomyocytes.

### 3.10 Verification of I_Ca-L_

**FIGURE 9:**
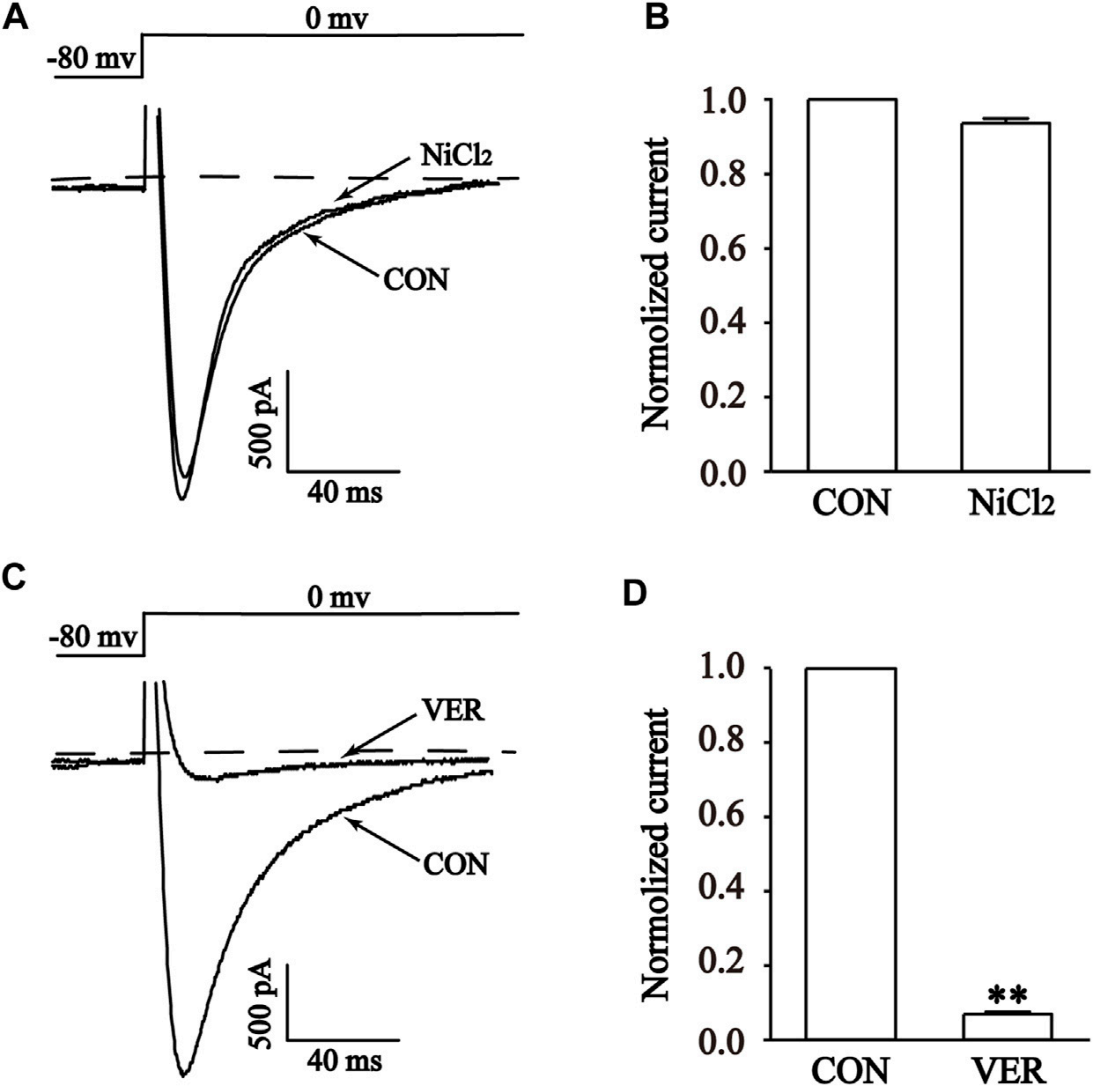
Validation of I_Ca-L_ in isolated cardiomyocytes. Representative curves **(A,C)** and combined data **(B,D)** for steady-state activation of I_Ca-L_ before and after using 1 μM Ver and 100 μM NiCl_2_. Mean ± SEM, *n* = 10. Each group has 10 cells from 10 rat hearts. **p < 0.01 versus CON group.

The high sensitivity of T-type Ca^2+^ channels to Ni^2+^ makes NiCl_2_ a commonly employed specific blocker for T-type Ca^2+^ channels. Furthermore, it exhibits near-complete inhibition of the T-type calcium current while exerting minimal impact on the L-type calcium current (Lee et al., 1999). NiCl_2_ was used to investigate its effect on the current at a concentration of 100 μM. Interestingly, the current was not inhibited by 100 μM NiCl_2_, indicating that the recorded current was not a T-type Ca^2+^ current (Figures 9A, B) (Guan et al., 2015). In contrast, 1 μM VER almost completely blocked the current (*p* < 0.01), suggesting that the recorded current was I_Ca-L_ (Figures 9C, D) (Zhu et al., 2016).

### 3.11 Inhibition of CYTA on I_Ca-L_

**FIGURE 10:**
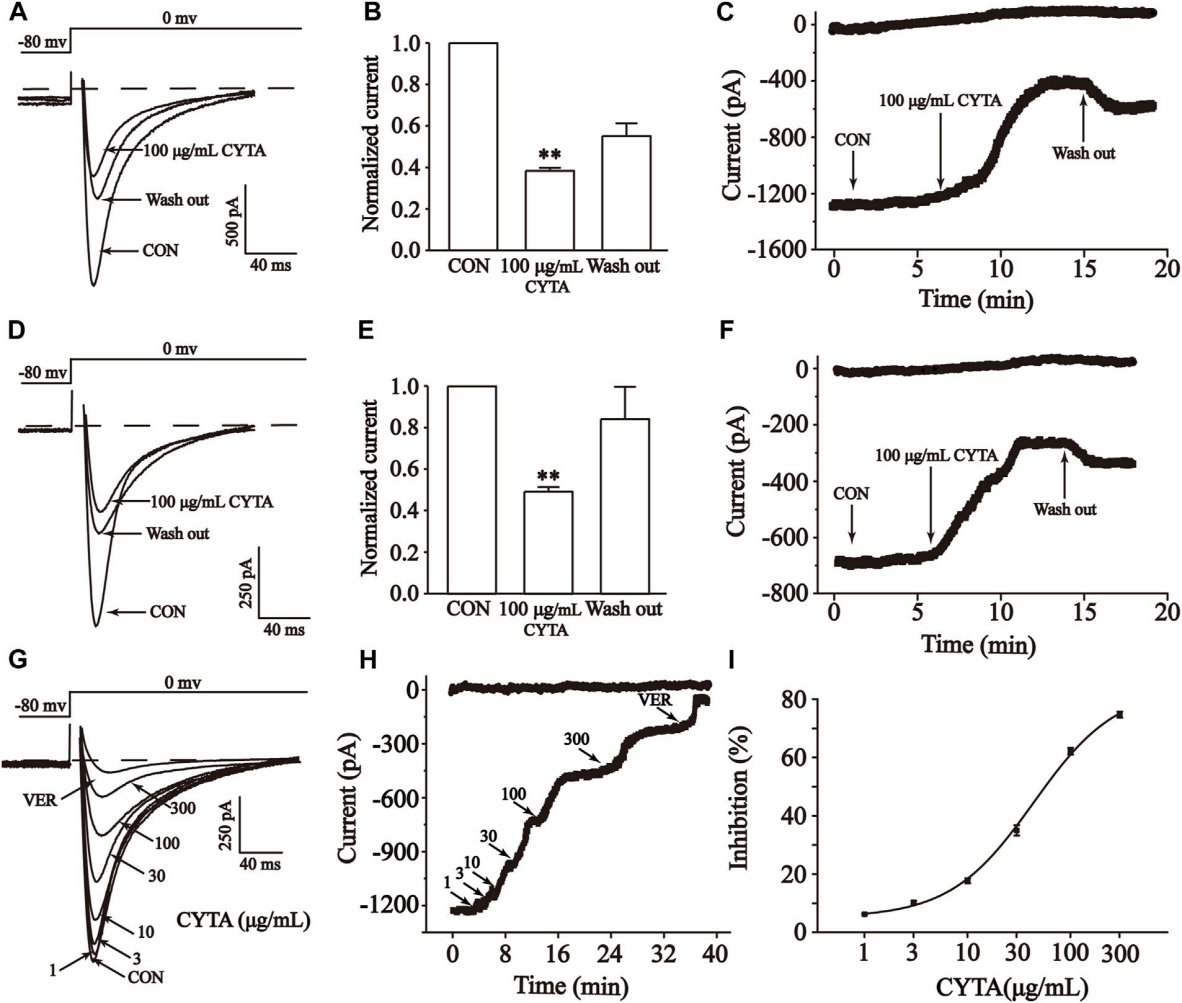
Effects of CYTA on I_Ca-L_ in isolated cardiomyocytes. Effects on normal **(A–C)** and ischemic cardiomyocytes **(D–F)** I_Ca-L_ were documented under CON, CYTA (100 μg/mL) and elution conditions **(A,D)** Representative examples of I_Ca-L_, **(B,E)** summarized data and **(C,F)** time constant. Typical trajectory **(G)**, time constants **(H)** and dose versus percentage inhibition curves **(I)** for I_Ca-L_ recorded at CON and 1, 3, 10, 30, 100 and 300 μM CYTA and 1 μM VER conditions. Mean ± SEM, *n* = 10. Each group has 10 cells from 10 rat hearts. **p < 0.01 for CON group.

The results demonstrate that CYTA at a concentration of 100 μg/mL inhibited I_Ca-L_ in both normal and ischemic rat ventricular myocytes, resulting in inhibition rates of 61.60% ± 1.49% and 50.79% ± 2.26%, respectively (*p* < 0.01). Furthermore, following the administration of CYTA, extracellular fluid was used for washout, and the currents were partially restored to their pre-administration levels. This suggests that the inhibitory effect of CYTA on I_Ca-L_ is reversible (Figures 10A–F).

The results presented in these figures show a sequential increase in the inhibitory effect of CYTA on I_Ca-L_ at concentrations of 1, 3, 10, 30, 100, and 300 μg/mL, resulting in inhibition rates of 2.12% ± 0.30%, 6.50% ± 0.56%, 14.45% ± 0.89%, 33.01% ± 1.95%, 62.17% ± 1.23%, and 75.65% ± 1.13%, respectively. This indicates a concentration-dependent effect of CYTA on I_Ca-L_, with a half inhibition rate (IC50) of 43.60 ± 8.61 μg/mL (Figures 10G–I).

### 3.12 Effects of CYTA on I_Ca-L_ current-voltage (I-V) curves in ventricular myocytes

**FIGURE 11:**
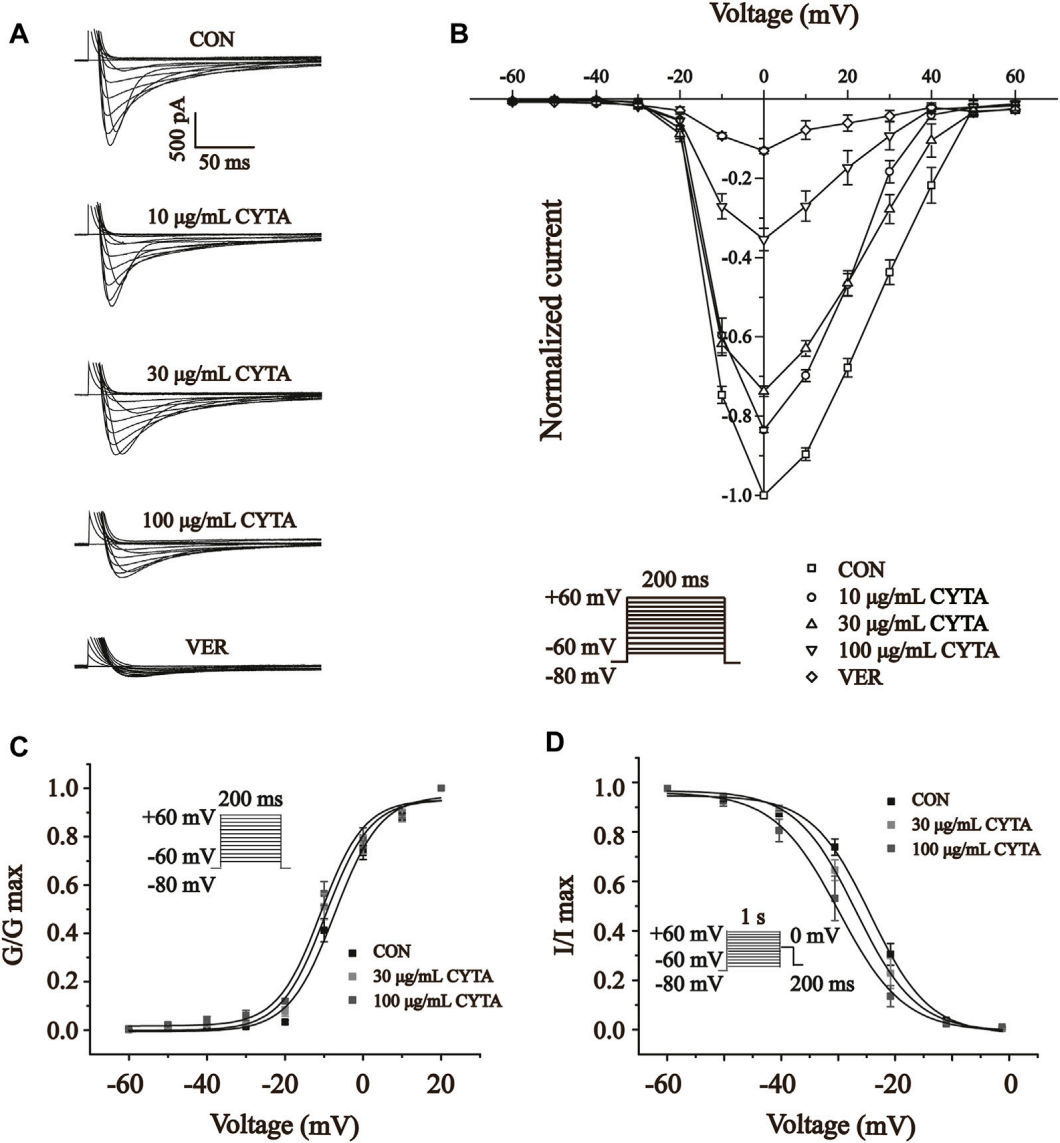
Effects of CYTA on I_Ca-L_ current-voltage relationships (I-V curves) and I_Ca-L_ steady-state activation and inactivation curves in isolated cardiomyocytes **(A)** Representative recording plots of I_Ca-L_ recorded in isolated cardiomyocytes under CON, CYTA (10, 30 and 100 μg/mL) and VER (1 μM) conditions. **(B)** I-V relationship curves plotted at three consecutive concentrations of CYTA (10–100 μg/mL) and VER (1 μM) were given. Voltage Steady-state activation **(C)** and inactivation curves **(D)** of I_Ca-L_ in ventricular myocytes before and after administration of different concentrations of CYTA (30 and 100 μg/mL). Mean ± SEM, *n* = 10. Each group has 10 cells from 10 rat hearts.

Figure 11A shows the effect of CYTA (10, 30 and 100 μg/ml) and VER on the relationship between the I-V curves. As shown in Figure 11B, CYTA shifts the I-V curve in a concentration-dependent manner. At −20 mV, the amplitude of I_Ca-L_ began to increase. In addition, the reversal potential of I_Ca-L_ and the I-V curve did not change significantly.

### 3.13 Effects of CYTA on steady-state activation and inactivation of I_Ca- L_

The results presented in Figures 11C, D indicate that CYTA at concentrations of 30 and 100 μg/mL did not significantly affect the steady-state activation-deactivation curve of I_Ca-L_. The calculated values for V_1/2_/slope (k) of the activation curves were −7.29 ± 1.14 mV/5.87 ± 0.99 mV, −9.64 ± 1.22 mV/5.70 ± 1.11 mV, and −10.84 ± 1.14 mV/5.56 ± 1.04 mV for 0, 30, and 100 μg/mL CYTA, respectively. Similarly, the V_1/2_/slope (k) of the inactivation curves were −23.61 ± 0.73 mV/5.40 ± 0.23 mV, −29.25 ± 0.72 mV/3.97 ± 0.24, and −29.89 ± 0.11 mV/5.81 ± 0.69 mV, respectively.

### 3.14 Effects of CYTA on contractility and temporal parameters

**FIGURE 12:**
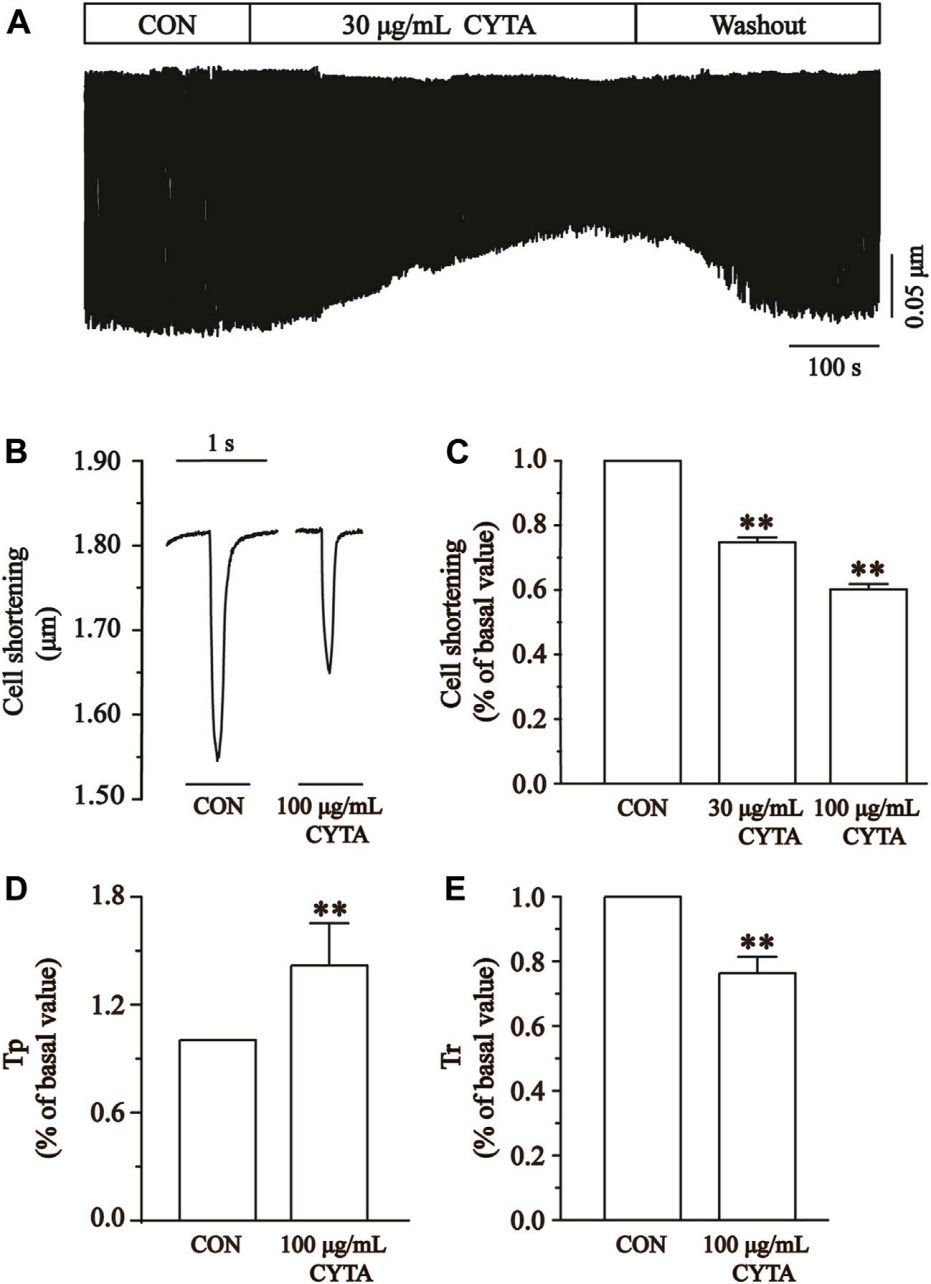
Effects of CYTA on cell contraction and time parameters of cell contraction in isolated cardiomyocytes. **(A)** The time progress of cell shortening was recorded in the existence or inexistence of 30 and 100 μg/mL CYTA. **(B)** Representative traces of cell shortening. **(C)** Statistical analysis of 30 and 100 μg/mL CYTA on cell shortening. **(D,E)** Statistical analysis results of Tp and Tr in the CON and 100 μg/mL CYTA. Mean ± SEM, *n* = 8. Each group has 8 cells from 8 rat hearts. **p < 0.01 versus CON group.

Cell contractility was assessed at CYTA concentrations of 30 and 100 μg/mL, as depicted in Figures 12A, B. Figure 12C demonstrates that these concentrations of CYTA resulted in the inhibition of cell contraction by 25.27% ± 1.55% and 39.83% ± 1.68%, respectively (*p* < 0.01). Notably, the administration of extracellular fluid for washout partially restored cell contraction, indicating a reversible inhibitory effect of CYTA on ventricular myocyte contractility.

The time to 50% peak (Tp) is an important measure of cell contraction or Ca^2+^ elevation, while the time to 50% of baseline (Tr) is crucial for assessing cellular relaxation or Ca^2+^ reabsorption. Figures 12D, E show that both Tp and Tr were significantly lower in the CYTA group compared to the CON group (*p* < 0.05).

### 3.15 Effects of CYTA on Ca^2+^ transients

**FIGURE 13:**
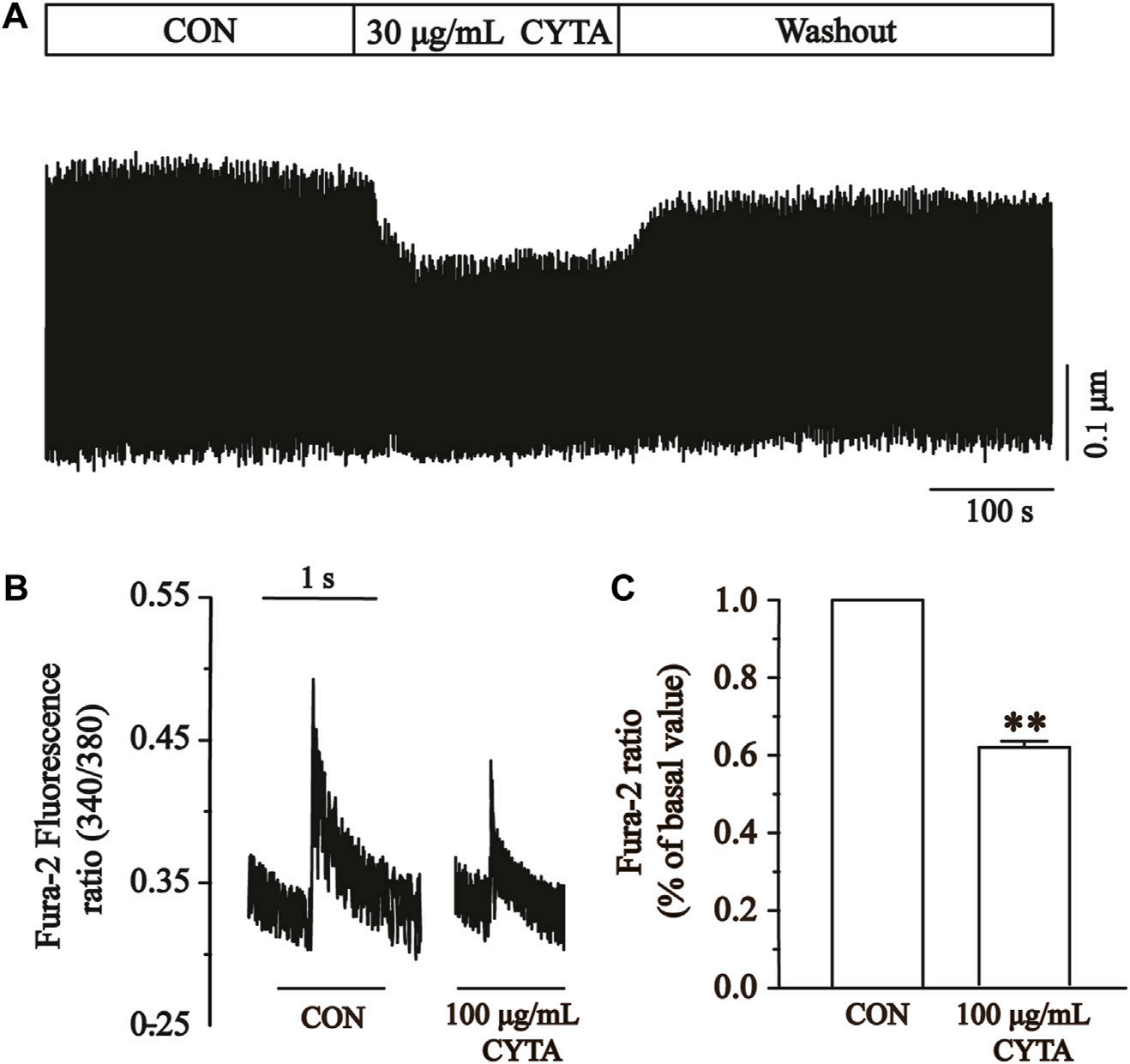
Effects of CYTA on Ca^2+^ transients in ventricular myocytes in isolated cardiomyocytes. Ca^2+^ transients recorded **(A)** in isolated cardiomyocytes in the presence or absence of CYTA (100 μg/mL) and representative trajectories **(B)** and summary data **(C)**. Mean ± SEM, *n* = 6. Each group has 9 cells from 9 rat hearts. **p < 0.01 versus CON group.

Figure 13A illustrates the impact of CYTA (100 μg/mL) on the peak change in Ca^2+^ transients. In Figure 13B, it is evident that a concentration of 100 μg/mL CYTA significantly inhibits the peak Ca^2+^ transient. Furthermore, Figure 13C demonstrates that CYTA at a concentration of 100 μg/mL resulted in a notable inhibition of the peak amplitude of cellular Ca^2+^ transient, with an inhibition rate of 38.48% ± 4.19% (*p* < 0.01).

## 4 Discussion

**FIGURE 14:**
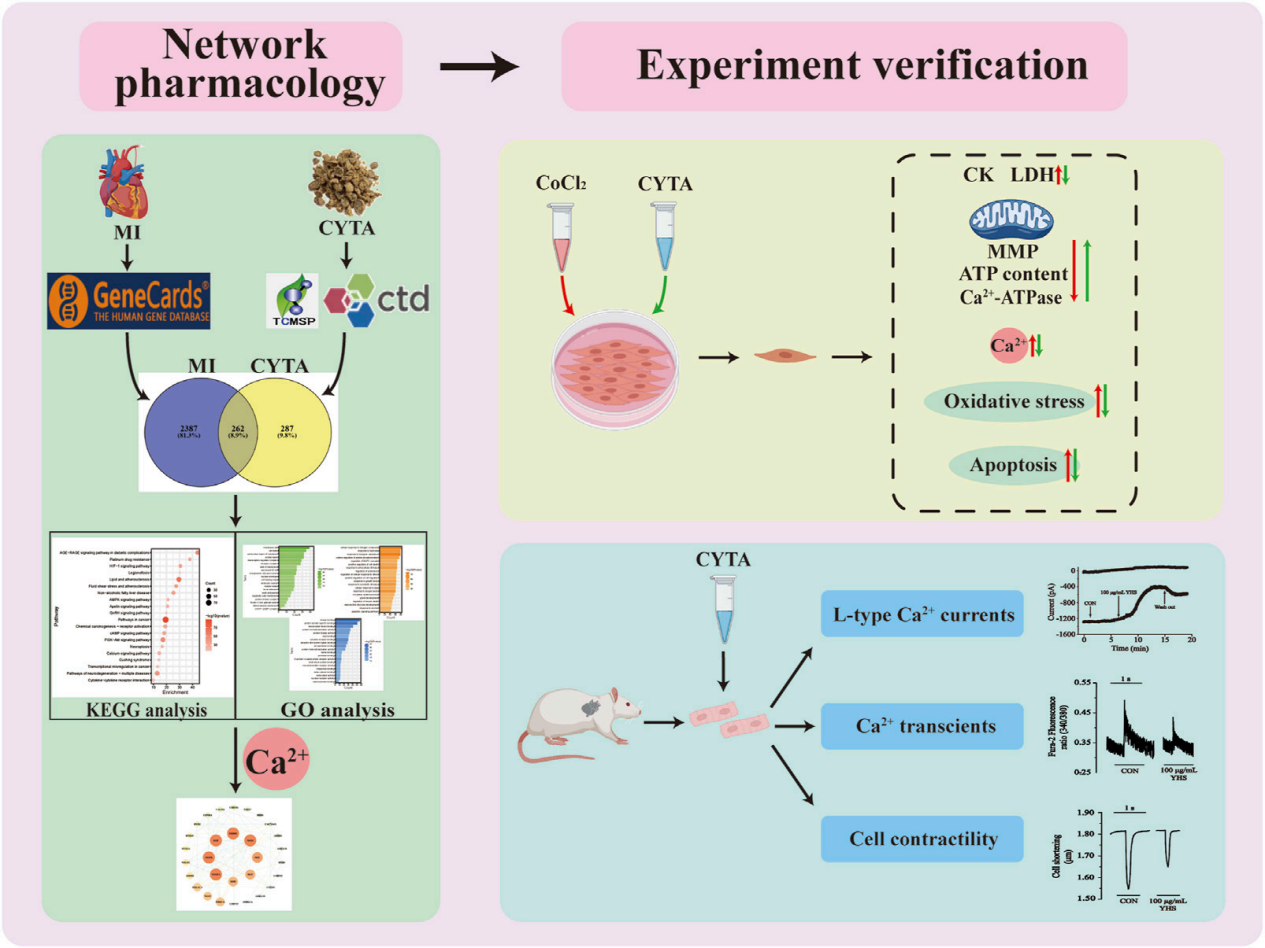
Workflow diagram for the discovery of potential mechanisms for CYTA against MI.

Repeated or prolonged MI leads to non-adaptive remodeling of cardiomyocytes and expansion of extracellular matrix, which ultimately leads to dilation of cardiac chambers and systolic dysfunction, inducing diseases such as coronary heart disease and heart failure. Despite the increasing research on the prevention and treatment of IHD in recent years, IHD maintains a high mortality and morbidity rate. This condition remains a significant burden on individuals and healthcare resources worldwide (Severino et al., 2020). This study first utilized network pharmacology to explore the potential targets of action of CYTA in the treatment of MI, followed by experimental validation with H9c2 cells and acutely isolated ventricular myocytes in rats (Figure 14). Initially, it was demonstrated that CYTA could protect H9c2 cells from hypoxic injury, and the protective mechanism might be related to the inhibition of oxidative stress, apoptosis, and mitochondrial dysfunction. Most importantly, our findings suggest that CYTA safeguards the heart by inhibiting L-type Ca^2+^ channels (LTCCs), which consequently reduces cellular contraction and prevents Ca^2+^ overload.

*Yanhusuo* is a perennial herb from the poppy family and is commonly used in clinical practice as a traditional Chinese medicine (Li et al., 2022). It has been the subject of various pharmacological studies, which have reported its therapeutic effects on numerous cardiovascular diseases. Bi et al. (2021) used data mining to confirm that the frequency of *yanhusuo* in clinical prescriptions for the treatment of coronary artery disease in Chinese medicine is higher than 60%. In addition, previous studies have shown that *yanhusuo* extract and its constituents such as tetrahydropalmatie have the ability to reduce left ventricular end-diastolic pressure, enhance cardiac ejection function in rats, improve cardiac function, and significantly reduce the size of myocardial infarction, as well as having the ability to mitigate myocardial infarction with antiarrhythmic effects (Ling et al., 2006; Wu et al., 2007; Tian et al., 2020; Han et al., 2022). However, there are no studies exploring the effects of CYTA on LTCCs and contractility of cardiomyocytes at the electrophysiologic level. It is well known that the membrane clamp is the gold standard for examining cellular electrophysiology. Currently, based on the electrophysiological method, numerous studies have been conducted on natural agents that can treat MI. For example, scholars such as Han et al. (2022); Guan et al. (2015) demonstrated that total flavones from *Acanthopanax senticosu*s and *ginseng* total saponin have therapeutic effects on MI by inhibiting Ca^2+^ influx, respectively. Therefore, the present study is based on this to develop the experimental design.

Using the principles and techniques of modern network pharmacology, our study revealed that the therapeutic effects of CYTA on MI are attributed to its multi-component, multi-target, and multi-signaling pathway properties. Initially, we identified 46 alkaloid components of CYTA (Table 2) through TCMSP and reference literature. Subsequently, we obtained 549 potential targets of CYTA (Figure 1) and 2649 MI-related targets. The intersection of these targets resulted in 262 cross-targets for further analysis (Figure 2A). Among the KEGG results, we specifically focused on the calcium signaling pathway as it appeared to be important in CYTA’s efficacy against MI (Figure 3B). Consequently, we extracted and demonstrated 30 targets involved in the calcium signaling pathway (Figure 2C). Notably, CALM2, a calcium ion signal transducer protein, plays a role in activating CALM2 through calcium, subsequently regulating multiple signaling pathways (Boczek et al., 2016). Additionally, CACNA1S is recognized as one of the components of LTCCs (Kessi et al., 2021). Moreover, GO enrichment analysis revealed that the potential targets of CYTA could influence BPs related to MI, such as anti-oxidative stress and anti-apoptosis (Figure 3A).

Therefore, to investigate the effects of CYTA on cardiac oxidative stress injury, cell apoptosis, and calcium signaling regulation, we conducted experiments using both an H9c2 cell hypoxia model and a rat MI model. The CoCl_2_ is a widely used and recognized chemical hypoxia simulation reagent. Co^2+^ can replace dried ammonia acyl of CoCl_2_ hydroxylase (PHD) of Fe^2+^, thereby enabling PHD inactivation. PHD plays a key role in the relationship between oxygen concentration and hypoxia-inducible factor (HIF) degradation. In addition, the presence of CoCl_2_ can stabilize HIF under normoxic conditions and this stability can be maintained for several hours, which is not achieved under hypoxia-induced hypoxia. Thus, the model allows us a time window to manipulate and analyze samples under normoxic conditions (Munoz-Sanchez and Chanez-Cardenas, 2019). The optimal concentration of CoCl_2_ for inducing hypoxia was determined using the CCK-8 method (Figure 4A). The measurement of CK and LDH release has been widely utilized as an adjunct method to verify myocardial damage (Long et al., 2012). Our results demonstrated that CYTA effectively protected H9c2 cells from CoCl_2_-induced damage, as evidenced by the decreased release of CK and LDH (Figures 4E, F).

When cardiomyocytes experience hypoxic damage, the primary mechanisms involved are the increased production of ROS, intracellular Ca^2+^ overload, and impaired mitochondrial energy synthesis (Paradies et al., 2018). In recent years, with the in-depth research on the mechanism of myocardial ischemic injury, it has been found that large amount of ROS generation and its triggered lipid peroxidation reaction is one of the main mechanisms of myocardial ischemic injury (Neri et al., 2015; Han et al., 2022). Hypoxia leads to decreased ATP production and the entry of Ca^2+^ into cells, activating Ca^2+^-dependent protein hydrolases, which convert xanthine dehydrogenase into xanthine oxidase. Consequently, a substantial amount of ROS is generated (Tsutsui et al., 2011). ROS reacts with unsaturated fatty acids, resulting in the production of lipid peroxides that further decompose into malondialdehyde (MDA). MDA combines with proteins containing free amino groups, phospholipid phosphoethanolamine, and nucleic acids (Zorov et al., 2014). This process leads to the formation of Schiff bases between MDA and free amino acids, such as proteins, phospholipid phosphoethanolamine, and nucleic acids, causing cross-linking between biomolecules.

These reactions ultimately result in protein denaturation, damage to the integrity of the myocardial cell membrane, increased membrane permeability, and the influx of extracellular Ca^2+^ into the intracellular space, leading to Ca^2+^ overload (Toescu, 2004; Wang et al., 2023). The accumulated intracellular Ca^2+^ can be absorbed by the sarcoplasmic reticulum and mitochondria, resulting in significant energy consumption. Additionally, the entry of Ca^2+^ into mitochondria can disrupt oxidative phosphorylation, thereby interfering with energy metabolism and directly reducing ATP production (Stanley, 2004). Therefore, the generation of ROS can induce cellular damage, while the damage to the cell membrane, alterations in the tissue microenvironment, and intracellular Ca^2+^ overload further accelerate cellular damage through a series of mechanisms (Barry, 1987).

It cannot be ignored that antioxidant enzymes such as CAT, SOD, GSH-Px play an important role in the process of cellular oxidative stress caused by ROS. CAT, also known as catalase, is an enzymatic scavenger that catalyzes the decomposition of H_2_O_2_ (Goyal and Basak, 2010). Another essential enzyme involved in scavenging free radicals is SOD, while MDA serves as an indicator of lipid peroxidation intensity (Tsutsui et al., 2011). GSH-Px catalyzes the decomposition of hydrogen peroxide within cells, reducing the peroxidation of polyunsaturated fatty acids in cell membranes by scavenging peroxides and hydroxyl radicals generated during cellular respiratory metabolism (Ferrari et al., 2004; Hall et al., 2009). The findings of our study revealed that CYTA reduced intracellular ROS levels and MDA content while increasing the activities of SOD, GSH-Px, and CAT (Figure 5). These results indicate that CYTA can counteract the oxidative stress damage in H9c2 cells through multiple pathways and improve the cellular antioxidant capacity.

During myocardial hypoxia, mitochondrial oxidative phosphorylation was impeded, leading to impaired ATP synthesis. In severe cases, mitochondrial swelling, crag disintegration, membrane fragmentation, and matrix spillage occurred, causing irreversible damage and triggering apoptosis (Bernardi and Di Lisa, 2015; Bernardi et al., 2015). Therefore, this study examined intracellular ATP content, Ca^2+^-ATPase activity, mitochondrial damage, and apoptosis. The results demonstrated that CYTA not only significantly improved intracellular ATP content and Ca^2+^-ATPase activity (Figures 6C, D) but also reversed mitochondrial damage (Figures 6A, B) and apoptosis (Figure 7) induced by hypoxia. These findings suggest that CYTA possesses antioxidant and anti-apoptotic effects on cardiomyocytes.

Ca^2+^ is a crucial messenger that regulates various BPs *in vivo*, including myocardial excitation-contraction coupling (Hofmann et al., 2014). Therefore, maintaining Ca^2+^ homeostasis is essential for normal cardiac physiological function (Barry and Bridge, 1993). On the one hand, elevated intracellular calcium ions directly induce myocardial contraction (Bai et al., 2005); On the other hand, they also participate in regulating several calcium-dependent protease activities, serving as second messengers (Hofmann et al., 2014).

There are two types of calcium channels present in cardiomyocytes: voltage-gated calcium channels and calcium-releasing calcium channels. Voltage-gated calcium channels are categorized into T, L, N, P/Q, and R types based on their pharmacological and biophysical properties (Catterall, 2011). The low voltage-dependent calcium channels exclusively consist of the T-type, which becomes activated and inactivated at relatively low membrane potentials (−70 to −60 mv) with a short duration. The high voltage-activated calcium channels encompass the L, N, P/Q, and R types, which activate at (−30 to −20 mv) and have a longer opening time (Catterall, 2011). Studies have reported that the VER exhibited significant anti-MI action in animal experiments and clinical studies. In addition, there are also studies using VER as a positive control group in animal experiment. Therefore, we chose VER as the positive control drug in the present (Han et al., 2022). Experimental results showed that the use of the specific I_Ca-L_ blocker VER was able to almost completely eliminate the current, indicating the function of LTCCs in ventricular myocytes (Figure 9).

LTCCs are proteins found in the cardiomyocyte membrane. Ca^2+^ enters the cell through LTCCs, triggering the release of stored Ca^2+^ in the sarcoplasmic reticulum via sarcoplasmic reticulum calcium release channels. This process plays a crucial role in the excitation-contraction coupling of the heart, facilitating the propagation of action potentials predominantly during rapid depolarization. Moreover, it contributes to the formation and maintenance of the plateau phase of the myocardial action potential (Yamaoka and Kameyama, 2003).

Therefore, the effects of CYTA on LTCCs and cardiomyocyte contractility were explored in this research. The results showed that CYTA had a reversible inhibitory effect on LTCCs in both normal and ischemic rats (Figures 10A–F), and this inhibitory effect was concentration-dependent (Figures 10G–I). This demonstrated that CYTA had a calcium channel antagonist-like effect and was able to reduce Ca^2+^ entry into the cells. Furthermore, the relationship between CYTA and I_Ca-L_ I-V curves reveals that CYTA can concentration-dependently suppress I_Ca-L_ peak current density while the activation and peak potentials remain unchanged. Consequently, this prolongs the action potential time course in cardiac myocytes, attenuates the rate of Ca^2+^ inward flow, and reduces myocardial contractility (Figure 11). Figure 11 indicates that CYTA does not significantly affect I_Ca-L_ activation and inactivation kinetics, suggesting that CYTA does not alter the rate of I_Ca-L_ activation and inactivation in rat ventricular myocytes.

When LTCCs are activated, it stimulates calcium influx, resulting in increased myocardial mechanical contraction and Ca^2+^ overload. The excessive contraction of cardiomyocytes leads to elevated oxygen consumption, potentially causing myocardial dysfunction and even cardiomyocyte death (Allen and Orchard, 1987; Crystal et al., 2013). Thus, inhibiting LTCCs can alleviate Ca^2+^ overload and hypercontractility of the myocardium.

The results indicate that CYTA effectively inhibits cardiomyocyte contractility (Figures 12A–C) and Ca^2+^ transients (Figure 13). Tp and Tr are critical temporal parameters that represent the rate of cell contraction and relaxation, respectively (Zhu et al., 2016). In conclusion, this study demonstrates that CYTA at a 100 μg/mL concentration significantly inhibits Tp and Tr (Figures 12D, E). These findings provide evidence that CYTA can hinder Ca^2+^ influx into cardiomyocytes by targeting LTCCs, reflecting the fact that CYTA has characteristics similar to those of antagonists of LTCCs. These findings all suggest that CYTA has potential for the treatment of MI.

Some limitations of this study should be considered. Our network pharmacology analysis revealed that SCN5A, KCNMA1, and KCNH2 are equally potential targets for MI treatment with CYTA. These targets are significant members of cell surface sodium and potassium channels (Bowlby et al., 2008; Li et al., 2018; Bailey et al., 2019). Apart from Ca^2+^ channels, the myocardial cell membrane also contains K^+^ channels, Cl^−^ channels, and Na^+^ channels, all of which play crucial roles in maintaining the normal physiological functions of the heart. The experimental subjects in this study consisted of cells isolated from the internal environment and H9c2 cells, thereby limiting the ability to fully replicate the complex physiological milieu *in vivo*. The H9c2 cells, despite sharing similar physiological and biochemical characteristics with primary cardiomyocytes, still exhibit distinctions in terms of cellular morphology, contractility, and gene expression (Zheng et al., 2021). Different species of animal experiments are needed to verify the protective effect of CYTA on MI. Electrophysiological studies from multiple directions, such as action potential duration, ion channel kinetics and the influence of membrane potential, should be carried out, which will deepen the understanding of CYTA. Therefore, the study of other electrophysiological aspects of CYTA will be an important area for future research and cannot be ignored. In the future, on the basis of the results of this study, the CYTA findings can be continuously verified in different experimental models to make the results reproducible and robust. And promote the transformation of scientific research to the clinical push CYTA speed up into clinical studies, clinical strategy for treatment of IHD to provide more.

## 5 Conclusion

In summary, the present study demonstrates that CYTA has a significant protective effect against MI, and its protective mechanism may be related to attenuation of oxidative stress, improvement of mitochondrial function, reduction of cardiomyocyte apoptosis, and maintenance of Ca^2+^ homeostasis through LTCCs. The results obtained from this series of experiments using CYTA in the treatment of myocardial infarction provide new insights for future research and development of drugs for the treatment of IHD.

## Data Availability

The original contributions presented in the study are included in the article/Supplementary Material, further inquiries can be directed to the corresponding authors.
